# Inflammatory Effects of *Bothrops* Phospholipases A_2_: Mechanisms Involved in Biosynthesis of Lipid Mediators and Lipid Accumulation

**DOI:** 10.3390/toxins13120868

**Published:** 2021-12-04

**Authors:** Vanessa Moreira, Elbio Leiguez, Priscila Motta Janovits, Rodrigo Maia-Marques, Cristina Maria Fernandes, Catarina Teixeira

**Affiliations:** 1 Departamento de Farmacologia, Escola Paulista de Medicina, Universidade Federal de Sao Paulo, Sao Paulo 04044-020, Brazil; vmoreira@unifesp.br; 2 Laboratório de Farmacologia, Instituto Butantan, Sao Paulo 05503-900, Brazil; leiguez@uni9.pro.br (E.L.); priscilajanovits@gmail.com (P.M.J.); rodrigo.marques@esib.butantan.gov.br (R.M.-M.); cristina.fernandes@butantan.gov.br (C.M.F.)

**Keywords:** *Bothrops* phospholipases A_2_, inflammation, lipid mediators, signaling pathways

## Abstract

Phospholipases A_2_s (PLA_2_s) constitute one of the major protein groups present in the venoms of viperid and crotalid snakes. Snake venom PLA_2_s (svPLA_2_s) exhibit a remarkable functional diversity, as they have been described to induce a myriad of toxic effects. Local inflammation is an important characteristic of snakebite envenomation inflicted by viperid and crotalid species and diverse svPLA_2_s have been studied for their proinflammatory properties. Moreover, based on their molecular, structural, and functional properties, the viperid svPLA_2_s are classified into the group IIA secreted PLA_2_s, which encompasses mammalian inflammatory sPLA_2_s. Thus, research on svPLA_2_s has attained paramount importance for better understanding the role of this class of enzymes in snake envenomation and the participation of GIIA sPLA_2_s in pathophysiological conditions and for the development of new therapeutic agents. In this review, we highlight studies that have identified the inflammatory activities of svPLA_2_s, in particular, those from *Bothrops* genus snakes, which are major medically important snakes in Latin America, and we describe recent advances in our collective understanding of the mechanisms underlying their inflammatory effects. We also discuss studies that dissect the action of these venom enzymes in inflammatory cells focusing on molecular mechanisms and signaling pathways involved in the biosynthesis of lipid mediators and lipid accumulation in immunocompetent cells.

## 1. Introduction

*Bothrops* spp. snakes are responsible for the majority of snakebites in Latin America. Envenomation by these snakes induces severe pathological alterations at the site of venom injection, characterized by an intense local inflammatory reaction associated with myonecrosis, pain, and hemorrhage, potentially leading to permanent tissue damage and disability [1,2,3,4,5,6]. The local inflammatory response to bothropic envenomation involves a set of events including an increase in vascular permeability, edema formation, hyperalgesia, the activation and infiltration of immunocompetent cells, and a complex network of inflammatory mediators driving the inflammatory response [2]. These events are caused by the activation of critical host defense mechanisms of the victims by direct and indirect actions of the toxins present in the snake venom. Proteomic studies of *Bothrops* snake venoms revealed that the phospholipases A_2_ (PLA_2_s) are ubiquitous components of these venoms and play an important role in the pathophysiology of envenoming by these snakes [7,8,9,10,11]. During envenomation, in addition to aiding prey digestion, these toxins have been described to display myotoxic, cytotoxic, hemolytic, hypotensive, anticoagulant, platelet aggregation inhibition/activation, and proinflammatory effects [12,13,14]. Regarding inflammation, in addition to exerting direct effects on cell membranes, these lipolytic enzymes can recruit mammalian PLA_2_s analogs of similar activity and trigger endogenous signaling systems that display and amplify the cell injury and host defense mechanisms triggered by the whole venom. This amplification is responsible for many acutely important consequences of *Bothrops* envenoming. Yet, as discussed below, studies with in vitro and in vivo models aiming to understand the inflammatory action of isolated venom PLA_2_s can contribute to the knowledge of the local inflammatory mechanisms induced by *Bothrops* snake venoms and those from the Viperidae family [3,15,16,17]. These studies might lead to the discovery of new therapeutic targets for a more efficient treatment of envenoming by viperid snakes, since the currently available antivenoms have low effectiveness to neutralize the local events promoted by their venoms [18]. Finally, due to the structural and functional similarities to mammalian group (G) IIA PLA_2_, the *Bothrops* PLA_2_s can constitute useful tools for studies on the roles of human GIIA PLA_2_s in inflammatory diseases. In this regard, the effectiveness of varespladib, an inhibitor of svPLA_2_s [19] in attenuating inflammatory events caused by Viperidae snake venoms has been demonstrated in mice experimental model [20].

### 1.1. Phospholipases A_2_

Phospholipases A_2_ constitute a group of enzymes with diverse biological functions, ranging from homeostasis and membrane remodeling to the generation of metabolites and second messengers involved in biological processes and signal transduction. These enzymes hydrolyze the acyl ester bond at the sn-2 position of phospholipids, generating free fatty acids, such as arachidonic acid (AA) and oleic acid, and lysophospholipids, such as lyso-PAF [21,22]. Currently, PLA_2_s are classified into close families, including secretory PLA_2_ (sPLA_2_), cytosolic phospholipase (cPLA_2_), and Ca^2+^-independent PLA_2_ (iPLA_2_); platelet activating factor acetylhydrolase (PAF); and lysosomal PLA_2_ (LPLA_2_), PLA/acyltransferase (PLAAT), α/β hydrolase (ABHD), adipose-PLA_2_ (AdPLA), and glycosylphosphatidylinositol (GPI)-specific PLA_2_ [23,24,25]. This classification of PLA_2_s is based on the cell location, amino acid sequence, molecular weight, presence of intramolecular disulfide bridges, calcium requirement, and catalytic activity, including the hydrolysis at the sn-2 position of glycerophospholipids [22,26]. The sPLA_2_ family is largely distributed in nature, and its components are classified into I, II, III, V, IX, X, XI, XII, XIII, and XIV groups. They are present in high concentrations in snake, bee, and wasp venoms and in several mammalian organs, cells, and pancreatic juice [21,27]. Among them, we highlight group I PLA_2_s, comprising the secreted enzymes found in the snake venoms of the Elapidae and Hydrophiidae families and in the pancreas secretion of mammals. Meanwhile, group II consists of sPLA_2_s found in snake venoms of the Viperidae family and in mammalian tissues and are expressed in inflammatory processes [13,28].

For a long time, the pathophysiological activities of sPLA_2_s were related exclusively to their enzymatic activity, capable of providing a substrate for the synthesis of second messengers and inflammatory mediators. However, alternative mechanisms of action have been associated with the ability of sPLA_2_ to interact with receptors or even specific domains present on cell membrane surfaces, such as heparan sulfate proteoglycans, which have already been described to be important for triggering the activation of other sPLA_2_s in the target cells [29,30,31,32]. The identification of sPLA_2_s binding proteins was initially achieved by Lambeau et al. (1989) [33], who used the sPLA_2_ from the *Oxyuranus scutelatus* snake venom, called OS2. Due to its prevalence in brain tissue, this protein was denominated an N (neuronal) type receptor. Then, through screening in various tissues and cells, for studies of other OS2 binding proteins, a second type of PLA_2_ receptor was described, present in rabbit skeletal muscles, called an M (muscle) type receptor [34]. The presence of this latter receptor was identified in several tissues and in neutrophils, monocytes, and human alveolar macrophages [35,36], but not in murine peritoneal macrophages [37]. The M-type phospholipase A_2_ receptor has a high degree of homology (~30%) to mannose receptors, a member of the lectin receptor family, constitutively expressed in macrophages [38,39]. Mannose receptors are involved in phagocytosis, antigen processing [29,38,40], and the production of inflammatory cytokines by macrophages [40,41,42]. Additionally, this receptor has been demonstrated to play a role in cell proliferation and AA release, via MAPKs (protein kinase activated by the mitogens family), induced by the group IB sPLA_2_s [43,44,45]. Furthermore, it has been revealed that group IIA sPLA_2_s can bind to mannose receptors and promote the release of IL-6 by human alveolar macrophages [46].

Investigations into the biological role of mammalian group IIA PLA_2_, also known as inflammatory PLA_2_, in the development of several pathologies of inflammatory and immunological origin have been described. Several studies have revealed that group IIA sPLA_2_s are present in high levels in rheumatoid arthritis [47,48,49,50], acute pancreatitis [51,52,53], septic shock [54,55], Crohn’s disease and ulcerative colitis [56,57,58], respiratory distress syndrome [59,60,61], bronchial asthma and allergic rhinitis [59,62], atherosclerosis [63,64], autoimmune diseases [65], and cancer [66,67,68,69]. These observations imply that both local and systemic inflammation are associated with the release of sPLA_2_s in vivo, thus raising the unclear question of the role of these PLA_2_s in inflammatory reactions. Additionally, it was found that proinflammatory cytokines, such as interleukin (IL)-1β, IL-6, and tumor necrosis factor alpha (TNF-α), induce, in a variety of tissues, the gene transcription of sPLA_2_s and the subsequent increase in their secretion, thus, supporting the hypothesis of the involvement of sPLA_2_s in inflammation [70,71,72,73]. In addition, sPLA_2_s activate intracellular signaling events in cells that participate in inflammatory processes, caused by the generation of second messengers, and the phosphorylation of kinases as of MAPK [74,75,76,77]. Thus, sPLA_2_s represent an important target for investigations regarding the mechanisms of inflammatory events.

### 1.2. Inflammation—General Concepts and Signaling Pathways

Inflammation is a response of body tissues to noxious conditions for restoring homeostasis, setting the stage for the healing and reconstitution of injured tissue. The acute inflammatory response to injury involves functional alterations of microvessels that occur early after injury and develop at varying rates. The major features of these alterations include transient vasoconstriction, followed by vasodilation, then, leakage of protein-rich fluid from the microcirculation leading to edema formation, and movement of phagocytic leukocytes into the site of injury followed by local pain [78]. Immunocompetent cells, such as neutrophils and monocytes, found in blood circulation are capable of rapidly infiltrating tissues; macrophages and dendritic cells reside within tissue and play key roles in tissue surveillance and antigen presentation [79,80]. The vascular and cellular reactions are triggered and highly regulated by chemical factors, called inflammatory mediators, which include cytokines, chemokines, vasoactive amines, and eicosanoids and are produced by plasma components or are released in close proximity to the injury by endothelial cells, tissue-resident leukocytes such as mast cells, and macrophages at the early stages, followed by infiltrated leukocytes. The effects of inflammatory mediators involve the engagement of specific receptors, which then display signaling pathways responsible for the immune response [81,82,83]. Parallel to changes in blood flow, the margination of leukocytes begins, and leukocytes adhere to the microvascular endothelium through rolling and firm adhesion, and then moving through the vascular wall into the interstitial tissue. The various steps in the leukocyte migration are regulated by different subsets of cell-adhesion molecules expressed by both leukocyte and endothelial cells [84,85]. Thereafter, the phagocytosis of offending agents by migrated leukocytes occurs, followed by a release of lysosomal enzymes and an increase in the oxidative metabolism in leukocytes, known as respiratory burst, resulting in the production of microbicidal agents, such as superoxide anion (O_2_^−^) and hydrogen peroxide (H_2_O_2_) [86,87]. Four major classes of receptor-mediated phagocytosis exist: receptors of complement that recognize complement-coated particles; Fcg receptors, which are constitutively active for phagocytosis of IgG-coated particles; mannose receptors that recognize mannose and fucose on the surface of pathogens; and β-glucan receptors that recognize β-glucans-bearing ligands [87]. Furthermore, receptors for the Fc portion of immunoglobulin G and mannose/fucose residues lead to the release of proinflammatory mediators and reactive oxygen [88].

Despite the stereotyped features associated with an inflammatory response, the signal pathways involved in this response are determined by the nature of the inflammatory trigger, the sensors that detect them, the inflammatory mediators released, and the tissue affected. A number of surface and cytosolic receptors expressed in innate immune cells can sense pathogen-associated molecular patterns (PAMPs), damage-associated molecular patterns (DAMPs), or venom-associated molecular patterns (VAMPs) with high sensitivity and specificity. The recognition of these molecules is achieved by interacting with pattern recognition receptors (PRRs) [89]. These sensing receptors include Toll-like receptors (TLRs) [90,91], C-type lectin receptors (CLRS), RIG-I-like receptors (RLRs), and nucleotide-binding domain leucine-rich repeat (NLRs) or nucleotide-binding and oligomerisation domain (NOD)-like receptors [92,93]. Among them, TLRs (TLR1-TLR10) are highly conserved transmembrane proteins that play an important role in recognizing microbial pathogens and endogenous damage molecules, thereby triggering the generation of signals for the production of proinflammatory proteins and cytokines, via cooperation of adaptor proteins (MyD88, TIRAP, TRIF, and TRAM) [94,95]. The recognition of specific molecular patterns by NLRs induces the oligomerisation of proteins in the cytosol, generating platforms called inflammasomes [96]. The inflammasome is a high molecular weight protein complex that elicits the activation of inflammatory caspases and the processing of pro-interleukin-1β (pro-IL-1β) and pro-IL-18, generating the mature biologically active cytokines and a rapid inflammatory form of cell death termed pyroptosis [97]. Several distinct inflammasomes have been identified, including NLR and the pyrin domain containing receptor 1 (NLRP1), NLRP3, and NLR; the caspase recruitment domain containing receptor 4 (NLRC4); and the AIM2-like receptors (ALR) family [98,99]. These receptors positively regulate genes related to inflammatory mediators, including cytokines and key enzymes in the biosynthetic pathways of lipid mediators known as eicosanoids [100].

The early induction of most inflammatory transcripts depends on networks of transcription factors whose activation is coupled to pathways of signal transduction. The nuclear factor-kappa B (NF-κB) is a major and the best-studied transcription factor of inflammatory response [101,102]. The binding of activated NF-κB to the nuclear promoter region of diverse inflammatory factors leads to the transcriptional activation and expression of inflammatory mediators and enzymes. Currently, the major signaling pathways involved in inflammation are mediated by the cascade phosphorylation of protein kinases, such as the mitogen activated kinases (MAPKs) encompassing ERK1/2, SAPK, c-Jun NH2-terminal or JNK and p38MAPKs, phosphatidylinositol 3 kinase (PI3K), protein kinase C (PKC), and protein tyrosine kinase (PTK). These kinases mediate distinct intracellular signaling pathways associated with cytokines production, cytokine receptors, growth factors, mobilization of intracellular Ca^2+^, and regulate a variety of functions of immunocompetent cells, including cell migration, phagocytosis, degranulation, respiratory burst, and programmed cell death [103,104,105,106,107,108,109,110,111,112].

An effective acute inflammatory response results in the removal of noxious factors followed by the resolution and repair stages [113]. The shift in inflammatory markers, including the switch from proinflammatory mediators to anti-inflammatory, resolution-inducing mediators (lipoxins, maresins, protectins, and resolvins), is vital for the change from inflammation to resolution [114]. This switch drives the transition from neutrophil to monocyte recruitment into the affected sites, resulting in the clearance of dead cells and other debris, assisted by the lymphatic system, and the initiation of tissue repair at the damaged site [115]. However, if the acute events are not properly controlled, the inflammatory response becomes detrimental to the host. Yet, if the acute response does not succeed in neutralizing the injurious stimulus, the resolution phase might not be appropriately induced, and a chronic inflammatory state may ensue, leading to several inflammatory-mediated diseases [116,117].

## 2. Inflammatory Effects of sPLA_2_ from *Bothrops* spp. Venoms

### 2.1. Bothrops svPLA_2_s Induce Inflammatory Events and Activate Defense Functions in Leukocytes

Phospholipases A_2_ of GIIA are major components of *Bothrops* spp. snake venoms and play important roles in the pathophysiology of envenoming by these snakes, including the inflammatory response. Although these enzymes conserve a chemistry and catalytic structure, the natural evolution of viperid venoms introduced alterations in their primary amino acid residues, generating various other biological and toxicological effects [118]. In general, the GIIA sPLA_2_s found in viperid snake venoms are classified as sPLA_2_s, known as ‘classic’, containing an amino acid aspartate at position 49 (Asp49) and catalyzing the hydrolysis of the ester bond at position sn-2 of glycerophospholipids in a Ca^2+^ dependent manner. Meanwhile, the other type of sPLA_2_s is described as ‘variant’ and contains a lysine at the same position 49 (Lys49), with or without low catalytic activity [119]. Such a substitution affects the ability of these proteins to bind to Ca^2+^, an essential cofactor for the stabilization of tetrahedral intermediate, which occurs in the catalytic reaction performed by the Asp49-sPLA_2_s [120]. Despite the lack of enzymatic activity, sPLA_2_s-Lys49 homologues maintain their damaging capacity in membranes through a mechanism that is not completely understood and independent of Ca^2+^ [12,121,122].

It has long been demonstrated that viperid sPLA_2_s are potent inductors of inflammation. Although they present differences in their catalytic activity, both viperid Asp49 and Lys49 PLA_2_ homologues are capable of inducing local inflammation in diverse experimental models [123,124,125]. As such, this group of enzymes is considered to be a major component responsible for the severe local edema in envenomings by *Bothrops* spp. The inflammatory response to venom PLA_2_s is characterized by edema and the marked infiltration of leukocytes into the site of toxin injection. Studies on the mechanism of local edema induced by viperid sPLA_2_s (svPLA_2_s) have demonstrated an early increase in vascular permeability and a local release of inflammatory mediators, which act synergistically to cause the initiation and development of the inflammatory events. Among these mediators are vasoactive amines, including histamine, serotonin, and substance P, as well as vasodilating prostaglandins. Yet, in vivo studies employing a pharmacological approach have demonstrated that antagonists of serotonin and H1 receptors of histamine reduced the progression of edema induced by both catalytically active and inactive variants of sPLA_2_ isolated from *B. asper* [126], *B. neuwiedii* [127], *B. jararacussu* [128,129], or *B. insularis* [130]. In support of these reports, the release of histamine and serotonin by mast cells was observed following stimulation with bothropic sPLA_2_s from *B. jararacussu* [128,131]. Consistent with this evidence, the contribution of mast cells to edema formation induced by viperid PLA_2_s was further observed in in vitro experimental models demonstrating the ability of sPLA_2_s isolated from *B. pirajai*, *B. jararacussu*, and *B. atrox* snake venoms to degranulate mast cells [125,128,131,132]. It is well known that upon activation, mast cells secrete and synthesize an array of inflammatory mediators, which trigger the earliest events of inflammation [133,134]. Moreover, the contribution of the catalytic activity for the edematogenic effect of the enzymatic active Asp49 from bothropic PLA_2_s was suggested by studies, revealing that the chemical modification of this sPLA_2_ by p-bromophenacyl bromide inhibited edema formation induced by these viperid PLA_2_s [126,127,128]. In addition, the role of lipid mediators, such as PAF and eicosanoids, for hyperalgesia induced by catalytic active venom PLA_2_s was highlighted by studies using a pharmacological approach [135]. These authors suggested that the enzymatic hydrolysis of membrane phospholipids played a role in these events by directly releasing the precursors of lipid mediators, such as lyso-PAF and AA.

As mentioned previously, leukocytes are central components of inflammation. An important cellular component exists in the inflammatory response to *Bothrops* sPLA_2_s. As such, the stimulatory effect of piratoxin-I, bothropstoxin-I, and -II from *B. pirajai* and *B. jararacussu*, respectively, on neutrophil chemotaxis was demonstrated in an in vitro experimental model [136]. This effect was revealed to involve the interaction of these sPLA_2_s with surface heparan binding sites of neutrophils, followed by the release of chemotactic mediator leukotriene B_4_ (LTB_4_) and PAF, and is independent of enzyme activity. Furthermore, the ability of these venom PLA_2_s to recruit an endogenous PLA_2_ through the activation of GTP-binding protein and PKC was added to the mechanisms by which they cause neutrophil migration [137]. Moreover, studies conducted using in vivo experimental models have demonstrated the ability of *Bothrops* sPLA_2_s to induce a marked influx of polymorphonuclear and mononuclear cells into the site of their injection, as demonstrated for both catalytic active and non-catalytic venom PLA_2_s, such as MT-III and MT-II from *B. asper* snake venom [123,138]. A similar effect was reported by other authors, investigating various sPLA_2_s isolated from different *Bothrops* spp. snake venoms, such as Bothropstoxin (BthTX)-I and BthTX-II; *B. jararacussu* [131], BnSP-7, a catalytically inactive PLA_2_ from *B. pauloensis* [139], BatroxPLA_2_ from *B. atrox* [140] and BJ-PLA_2_-I from *B. jararaca* [141] in in vivo experimental models. The sPLA_2_-induced leukocyte migration was linked to the upregulation of adhesion molecules, such as l-selectin, LFA-1, and CD18, which in turn was associated with the release of inflammatory cytokines IL-1β, IL-6, and TNF-α with chemotactic activity by resident leukocytes, primarily macrophages [123]. Cytokines, chemokines, and leukotriene B_4_ are among the major mediators regulating the expression of adhesion molecules and chemotaxis of leukocytes [142,143,144]. Consistent with this information, increased serum levels of IL-6, IL-1, and TNF-α induced by Bbil-TX from *B. bilineata* snake venom were observed in a mouse experimental model [145]. In addition, there are reports that two Lys49 PLA_2_s isolated from *B. mattogrossensis* (BmaTX-I and BmaTX-II) venom were able to induce the release of IL-1β by murine neutrophils in culture [146] and that BatroxPLA_2_, an acidic sPLA_2_ from *B. atrox* venom, induced the release of IL-6, PGE_2_, and LTB_4_ from murine macrophages in culture [140]. In this context, the involvement of inflammasomes in the production of IL-1β induced by *Bothrops* sPLA_2_s was recently investigated. The participation of NLP3 inflammasome via the activation of caspase 1 in the production of IL-1β induced by BthTX-I, a Lys49-PLA_2_ from *B. jararacussu* venom, injected into mouse gastrocnemius muscle was reported [147]. In addition, the participation of inflammasomes in BthTX-I-induced production of IL-1β was demonstrated in peritoneal macrophages. This effect was demonstrated to be dependent on caspase 1/11, ASC, and NLRP3 and was associated with the release of ATP and activation of P2X7 receptors [148]. Despite the importance of cytokines, chemokines, and eicosanoids in orchestrating the events of inflammation and the potent proinflammatory effects triggered by viperid sPLA_2_s, including those from *Bothrops* genus, a complete picture of the inflammatory mediators released by immunocompetent cells upon stimulus by *Bothrops* sPLA_2_s has yet to be further investigated. Moreover, the mechanisms involved in the production and release of these mediators and the possible crosstalk between them remain to be better clarified. Regarding the mechanisms involved in the biosynthesis of lipid mediators induced by *Bothrops* sPLA_2_s, the progress made is presented in this review as a separate item (Section 2.2).

It is well recognized that the activation of innate effector functions, such as phagocytosis, and the production of microbicidal substances in leukocytes are critical for host defense and tissue repair. Regarding phagocytosis, studies have demonstrated the activity of *Bothrops* sPLA_2_s to induce phagocytosis following the activation of distinct receptors in immune-competent cells. In this sense, it was demonstrated that MT-II and MT-III, isolated from *B. asper* snake venom, can directly stimulate phagocytosis by macrophages in culture. MT-II significantly increased phagocytosis mediated by all classes of receptors, whereas MT-III increased phagocytosis via only mannose and beta-glucan receptors. This suggests that although the catalytic activity of *Bothrops* sPLA_2_s is not an essential requirement for enhancing macrophage phagocytosis, it may drive the class of phagocytosis receptors involved in this process. Molecular regions distinct from the catalytic network are likely involved in this effect [138]. In addition, the signaling pathways mediating zymosan phagocytosis, induced by both MT-II and MT-III, were investigated, with a focus on lipid second messengers. This study demonstrated that whereas the effect of MT-III, catalytically active, was dependent on the activation of endogenous iPLA_2_, the effect of MT-II was dependent on both endogenous iPLA_2_ and cPLA_2_. Likewise, COX-2 and 5-LO-derived metabolites in addition to PAF were involved in the signaling events required for phagocytosis induced by both venom sPLA_2_s [138]. In line with these data, BaltTX-I, devoid of catalytic activity and isolated from *B. alternatus* snake venom, was reported to activate the phagocytosis of serum-opsonized zymosan by murine macrophages, indicating the involvement of complement receptors. In addition, the participation of PKC was demonstrated. Nonetheless, BaltTX-II, a catalytically active sPLA_2_ isolated from the same venom did not stimulate phagocytosis in macrophages, lending support to previous findings that the catalytic activity of *Bothrops* sPLA_2_s is not essential for the stimulation of phagocytosis via complement receptor [149]. In addition, the sPLA_2_s isolated from Panamanian *B. asper* snake venom, pMTX-III (catalytically active Asp49) and pMTX-II and -IV, two enzymatically inactive Lys49 isoforms, were described to induce phagocytosis via mannose receptor and superoxide production in macrophages [150]. The mechanisms underlying the differences between the catalytic and non-catalytic active *Bothrops* PLA_2_s, regarding the activation of phagocytosis in macrophages and the participation of distinct receptors in their effects, require further clarification.

Concomitantly with phagocytosis, there is an increase in the oxidative metabolism, also referred to as respiratory burst, in leukocytes. In this context, the literature reveals that viperid sPLA_2_s can trigger the respiratory burst in immunocompetent cells. In the first study describing the ability of *Bothrops* sPLA_2_ to induce the release of microbicidal agents, the authors demonstrated that MT-II and MT-III, isolated from *B. asper* snake venom, induced the release of H_2_O_2_ by macrophages, with MT-III being the more potent stimulator [151]. In agreement with this evidence, it has been demonstrated that BaltTX-I and BaltTX-II from *B. alternatus* snake venom induced superoxide production by macrophages in culture in a process mediated by PKC [149]. In addition, other authors have revealed that the three sPLA_2_s from *B. atrox* venom, namely BaTX-I, a Lys49 variant devoid of catalytic activity; BaTX-II, a catalytically active Asp49; and BaPLA_2_, an acidic Asp49 sPLA_2_ induced the release of the superoxide anion by the J774A.1 lineage macrophages in culture [152]. BaTX-I was the only sPLA_2_ able to stimulate complement receptor-mediated phagocytosis, but all studied sPLA_2_s could increase the macrophage lysosomal volume [152]. These data demonstrate the ability of *Bothrops* PLA_2_s to trigger the respiratory burst, which is an essential process for the elimination of harmful agents. Although the structural determinants of such an effect were not investigated, it is likely that neither the enzymatic activity nor the basic or acidic characteristic of PLA_2_ is essential for the activation of the respiratory burst.

**Figure 1:**
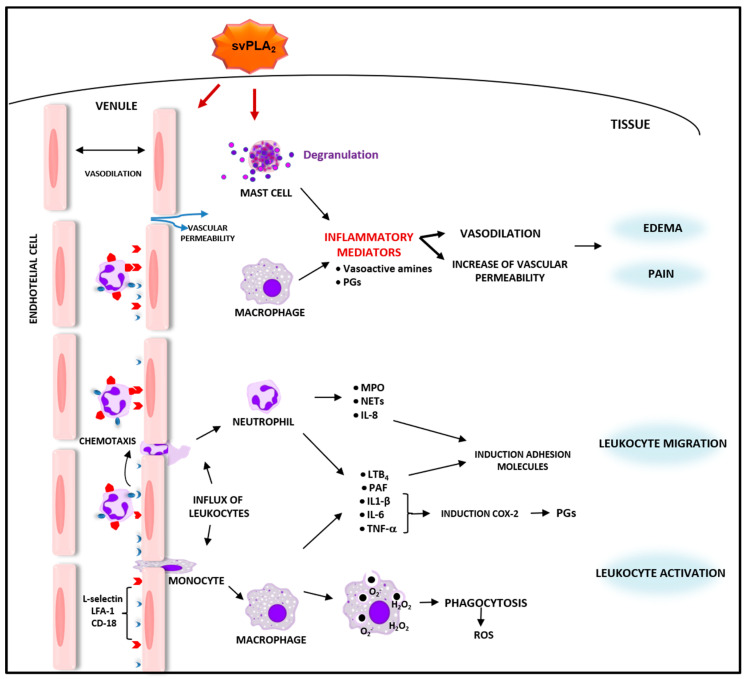
Scheme of inflammatory activities of svPLA_2_s. The svPLA_2_s induce inflammatory events, characterized by activation of innate immune cells and endothelial cells and release of several inflammatory mediators that interfere in the vascular dynamic. svPLA_2_s induce mast cells degranulation and activation of resident macrophages with release of inflammatory mediators such as prostaglandins (PGs), histamine, serotonin, and substance P, which lead to vasodilation, increase of vascular permeability, culminating in edema formation and pain. In addition, svPLA_2_s activate phagocytosis by macrophages and increase the local production of oxygen reactive species (ROS). Furthermore, svPLA_2_s, along with vascular alterations and produced inflammatory mediators, increase the expression of adhesion molecules such as LFA, CD-18 and L-selectin. These adhesion molecules, in turn, promote chemotaxis and leukocyte migration. The svPLA_2_s induce production of myeloperoxidase (MPO) and release of NETs by neutrophils. Both neutrophils and macrophages release proinflammatory mediators such as platelet-activating factor (PAF), IL-8, LTB_4_, IL-1β, IL-6, and TNF-α. These last three mediators are involved in the upregulation of COX-2 isoform, and release of PGs, thus amplifying the inflammatory response induced by svPLA_2_s.

An additional defensive strategy important for host defense is the neutrophil extracellular trap, or ‘NET’. The formation of NET (NETosis) occurs through the release of nuclear DNA, forming a sticky ‘net’ of extracellular fibers that can halt the dissemination of pathogens and toxins [153,154]. Despite its importance in the inflammatory response, little attention has been paid to the involvement of this defense mechanism in the effects of viperid sPLA_2_s. Yet, a report indicates that BaTX-II, an Asp49 PLA_2_ isolated from *B. atrox* snake venom, can activate human neutrophils in culture to produce hydrogen peroxide via the PI3K signaling pathway. Furthermore, this sPLA_2_ stimulated neutrophils to secrete MPO, NETs, and inflammatory mediators, including IL-1β, IL-8, and LTB_4_ [155]. Therefore, the activation of neutrophilic functions, including toxin trapping and inactivation, is likewise involved in the inflammatory response to *Bothrops* sPLA_2_s. Further studies are necessary to amplify the knowledge regarding the participation of NETs in inflammation induced by *Bothrops* spp. sPLA_2_s. Interestingly, in contrast to the reported ability of *Bothrops* sPLA_2_s to activate distinct inflammatory functions in leukocytes, a report revealed that CB (Crotoxin B), a catalytically active sPLA_2_ isolated from *Crotalus durissus terrificus*, which is a subunit of crotoxin complex [156,157], could, per se, display inhibitory effects in macrophage functions, including spreading and phagocytosis [158]. Such an inhibitory effect suggests an anti-inflammatory activity for this particular viperid sPLA_2_ [159]. In agreement with this idea, CB was reported to reduce the release of inflammatory cytokines, including IL-6 and TNF-α, and increase the release of PGE_2_ and lipoxin A4, both immunomodulatory lipid mediators, in dendritic cells [160]. A summary of the inflammatory activities of svPLA_2_s is illustrated in Figure 1. In Table 1, the svPLA_2_s-induced inflammatory responses are summarized according to the amino acid residue at position 49 and basic and acidic characteristics.

### 2.2. Influence of Bothrops svPLA_2_s on Pathways of Arachidonic Acid Metabolism

**Figure 2:**
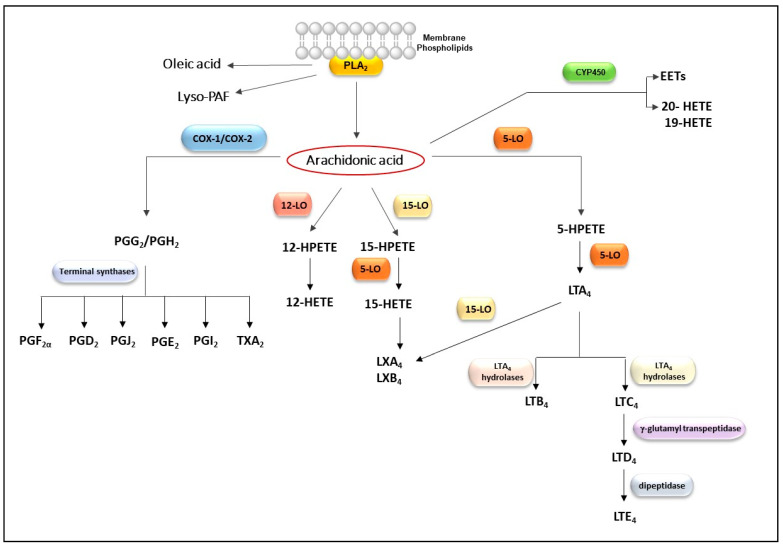
Scheme of arachidonic acid metabolism by several enzymatic pathways leading to production of bioactive lipid mediators. Abbreviations: (PLA_2_) phospholipase A_2_, (Lyso-PAF) lysophospholipid-platelet-activating factor, (COX-1) cyclooxygenase-1, (COX-2) cyclooxygenase-2, (5-LO) 5-lipoxygenase, (15-LO) 15-lipoxygenase, (12-LO) 12-lipoxygenase, cytochrome P450 (CYP450), (PGG_2_) prostaglandin G_2_, (PGH_2_) prostaglandin H_2_, (TXA_2_) thromboxane A_2_, (PGE_2_) prostaglandin E_2_, (PGD_2_) prostaglandin D_2_, (PGJ_2_) prostaglandin J_2_, (PGF_2α_) prostaglandin F_2_ alpha, (PGI_2_) prostacyclin, (15-HPETE) 15-hydroperoxyeicosatetraenoic, (15-HETE) 15-hydroxyeicosatetraenoic acid, (12-HPETE) 12-hydroperoxyeicosatetraenoic, (12-HETE) 12-hydroxyeicosatetraenoic acid, (5-HPETE) 5-hydroperoxyeicosatetraenoic, (5-HETE) 5-hydroxyeicosatetraenoic acid, (LTA_4_) leukotriene A_4_, (LTB_4_) leukotriene B_4_, (LTC4) leukotriene C_4_, (LTD_4_) leukotriene D_4_, (LTE_4_) leukotriene E_4_, (LXA_4_) lipoxin A_4_, (LXB_4_), lipoxin B_4_, (19-HETE) 19-hydroxyeicosatetraenoic acid, (20-HETE) 20-hydroxyeicosatetraenoic acid, (EETs) epoxyeicosatrienoic acids.

It is well established that sPLA_2_s play key modulatory roles in numerous cellular processes in physiological and pathological conditions by regulating the release of AA from membrane phospholipids [27,171]. It has long been recognized that the AA-derived lipid mediators are potent mediators of inflammation [83]. The AA is rapidly metabolized by several enzyme complexes, including cyclooxygenases (COX), lipoxygenases (LOX), and cytochrome P450 (CYP450). These enzymatic pathways promote the synthesis of oxygenated and bioactive products, generically called eicosanoids, which include prostaglandins (PG), leukotrienes (LT), hydroperoxyeicosatetraenoic acids (HPETEs), hydroxyeicosatetraenoic acids (HETEs), epoxides (EETs), and lipoxins (LX) [172,173,174,175,176,177,178]. A summary of the cascades involved in biosynthesis of eicosanoids is shown in Figure 2.

It is important to emphasize that COX-1 is a constitutive isoform present in most tissues and is responsible for generating PGs for diverse physiological functions [179,180,181,182]. In contrast, COX-2 is upregulated by inflammatory cytokines and growth factors [183,184] and is constitutively expressed in some tissues [185,186].

Regarding the production of inflammatory eicosanoids by *Bothrops* svPLA_2_, studies have demonstrated that the intraperitoneal injection of MT-III [187] and MT-II [162] in mice induced an early and transient release of PGD_2_, followed by a rapid and sustained release of PGE_2_. Likewise, in mice injected with BatroxPLA_2_ [140], from *B. atrox* snake venom, and BJ-PLA_2_-I from *B. jararaca* [141], an early release of PGE_2_ was observed. The in vivo experimental models of previous studies have revealed that *B. asper* sPLA_2_s induce the release of other eicosanoids, such as thromboxane A_2_ (TXA_2_) and LTB_4_ [123]. Moreover, an Asp49 svPLA_2_ from *B. atrox* venom [140] stimulated the production of LTB_4_, lipoxin, and PGE_2_.

PGE_2_ and PGD_2_ are important modulators of vasodilation, and PGE_2_ can potentiate an increase in vascular permeability, promoted by mediators of this phenomenon, with a consequent formation of edema [188,189]. Studies using pharmacological treatment with non-steroidal anti-inflammatory compounds were crucial in demonstrating the participation of these COXs-derived lipid mediators on edema [126,190] and hyperalgesia [135], induced by *B. asper* sPLA_2_s. In addition, studies demonstrating that MT-III and MT-II upregulated COX-2 protein expression in peritoneal leukocytes without altering the constitutive expression of COX-1 evidenced the ability of these venom PLA_2_s to influence downstream cyclooxygenase isozymes and suggested this as a mechanism by which these sPLA_2_s induced the production of prostaglandins [162,163]. Moreover, these findings suggested that the catalytic activity of these bothropic PLA_2_s did not contribute to the induction of PG biosynthesis, since MT-II, devoid of catalytic activity, caused the same effect.

In this regard, studies have demonstrated that the IκB phosphorylation inhibitor TPCK effectively prevented both MT-II- or MT-III-induced COX-2 expression, suggesting that the activation of NF-κB was critical for the induction of COX-2 expression by these bothropic svPLA_2_s. The involvement of NF-κB as the mechanism underlying this venom sPLA_2_s-induced upregulation of COX-2 expression was further confirmed by results that revealed the inhibition of the NF-κB nuclear translocation site, markedly reduced svPLA_2_s-induced COX-2 expression and, as a consequence, reduced PGE_2_ production by macrophages in culture [162,164].

**Figure 3:**
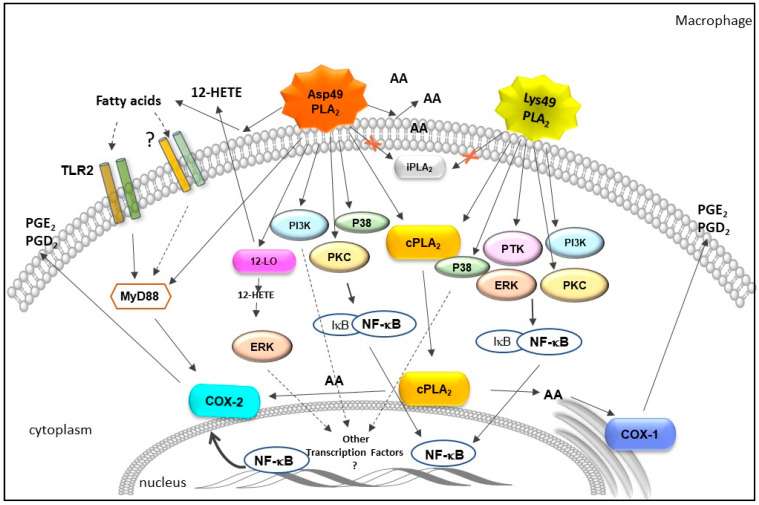
Schematic representation of signaling pathways stimulated by Asp49 and Lys49 PLA_2_s from *B. asper* snake venom to produce prostaglandins in macrophages. Asp49 PLA_2_ induces AA and fatty acids release from macrophage membrane. Free fatty acids can act as DAMPs and activate TLR2 or other TLRs (still unknown), via activation of adapter protein MyD88, leading to COX-2 protein expression and release of PGs, Asp49PLA_2_ upregulates the 12-LO pathway, culminating the release of 12-HETE. 12-HETE, in turn, activates the ERK1/2 pathway, leading to COX-2 protein expression and PG release, independent on NF-κB translocation. Asp49PLA_2_ also activates the signaling protein PI3K leading to COX-2 expression and production of PGs independent on NF-κB activation. Asp49 PLA_2_ also activates PKC and p38 MAPK pathways promoting COX-2 expression and production of PGs via NF-κB activation. In addition, Asp49PLA_2_ provides AA for activation of COX-1 activity which is followed by production of proinflammatory PGs. Asp49 and Lys49 PLA_2_s both produce PGs by pathways independent on iPLA_2_. Although both sPLA_2_ from bothropic venom produce PGs via crosstalk with cPLA_2_, only Lys49PLA_2_ induces COX-2 expression dependent on this cytosolic PLA_2_. Lys49PLA_2_ activates signaling pathways mediated by p38 MAPK, PTK, PKC, and ERK1/2. All these kinase pathways, except for p38 MAPK, are involved in NF-κB activation and COX-2 protein expression and PG production. Full arrows indicate actions already studied and demonstrated. Dotted arrows indicate hypothesized or unknown effects.

Studies employing mouse resident peritoneal macrophages or neutrophils in culture revealed that viperids sPLA_2_s induced a marked release of PGE_2_ in cell supernatants, accompanied by the release of AA [162,165,166,191]. These data support the results in vivo and serve as evidence that immune innate leukocytes, such as resident macrophages and neutrophils, are important sources of PGs under in vivo stimuli by sPLA_2_ from *Bothrops* spp. snake venoms. Interestingly, studies have demonstrated that the incubation of resident peritoneal macrophages with MT-II or MT-III significantly increased the concentration of AA [162,164]. Although the release of AA induced by the Asp49 sPLA_2_ was approximately 20 times greater than that induced by MT-II, it demonstrated that the catalytic activity of viperid sPLA_2_s was not an essential requirement for inducing COX-2 expression and PGE_2_ production. According to Kini and Evans (1995) [192], in mechanisms independent on catalytic activity, as in the case of MT-II, the interaction of sPLA_2_s to acceptor regions can cause the biological effect or interfere with the interaction of target proteins with their physiological ligands. Furthermore, some effects may result from combinations of both enzymatic and non-enzymatic mechanisms [192], leading to the activation of several signaling pathways; this should be considered when interpreting the effects of group IIA Lys49 svPLA_2_s. In this context, since crosstalk among sPLA_2_, cPLA_2_, and iPLA_2_s has been demonstrated to occur in several physiological and inflammatory conditions [193,194,195,196], the contribution of prey/victim cPLA_2_ and/or iPLA_2_ to the increased production of PGs and upregulation of COX-2 protein expression induced by svPLA_2_ variants was evaluated in diverse in vitro experimental models [162,165,187]. Thus, the pharmacological treatment of cells with the cPLA_2_ inhibitor but not the iPLA_2_ inhibitor decreased the release of AA and the production of PGE_2_ and PGD_2_ induced by svPLA_2_. In contrast, these pretreatments did not modify the MT-III-induced COX-2 expression but reduced the COX-2 expression induced by MT-II. These results demonstrate that cPLA_2_ is required for distinct actions of MT-II in the PG biosynthetic pathway in macrophages [162,187]. This is consistent with the reported functional cooperation between intracellular PLA_2_s and GIIA sPLA_2_ for PG biosynthetic responses in several other cell systems [171,197,198,199]. The role of cPLA_2_ as a key enzyme in supplying AA for COX-2-dependent PGs production is well established [171,200,201]. Taken together, the available data demonstrate that the Asp49 svPLA_2_s are functionally coupled with cPLA_2_, since prior activation of cPLA_2_ is required for MT-III to act with downstream enzymes for PG biosynthesis in macrophages and neutrophils [165,187]. Interestingly, the association of Lys49 sPLA_2_ with cPLA_2_, in addition to being important for the supply of AA for the production of PG, appears to modulate the transcription and protein expression of COX-2 inflammatory isoform. The mechanisms involved in the coupling between the venom GIIA sPLA_2_s and mammalian cPLA_2_ have yet to be investigated. One possibility is that GIIA svPLA_2_s activate cPLA_2_ by distinct signaling cascades that mimic the transducing mechanism conveyed by physiological activators of cPLA_2_, such as MAPKs, since this enzyme family is likewise important for the activation of NF-κB [202]. In line with this concept, the Asp49 svPLA_2_, MT-III, in addition to Lys49 PLA_2_, MT-II, from *B. asper* snake venom, were revealed to stimulate the phosphorylation of protein kinases, including the MAPKs, such as p38MAPK and ERK1/2 [167,169,171]. Moreover, other protein kinases, including PI3K, PKC, and PTK, were reported to be phosphorylated in macrophages stimulated by both MT-III and MT-II [162,164]. In this regard, studies have demonstrated that MT-III (Asp49 sPLA_2_) stimulates PKC and p38MAPK pathways to positively modulate PGE_2_ production and COX-2 expression via NF-κB, while MT-II (Lys49 PLA_2_) displays similar effects by activating PKC, ERK1/2, and PTK in murine peritoneal macrophages [167,169,171]. Since PTK is involved in the activation of MAPKs, which, in turn, are essential for cPLA_2_ activation, this signaling protein might be involved in the activation of cPLA_2_ by MT-II [202,203,204]. Furthermore, another pathway implicated in the release of PGE_2_ and the expression of COX-2 induced by MT-III was demonstrated to be independent of NF-κB activation. This pathway involved the activation of ERK1/2 by the 12-HETE pathway, the main product of 12-LO [166]. The involvement of another transcription factor in these MT-III-induced effects was suggested by the authors. Together, these findings reveal the variety and complexity of the mechanisms involved in the effects of svPLA_2_ leading to the generation of lipid mediators. The signaling molecules and pathways acting in an innate immune cell (macrophage) upon stimulus either by Asp49 or Lys49 svPLA_2_ are summarized in Figure 3.

Although the participation of the M-type PLA_2_ receptor or another type of interaction of *Bothrops* sPLA_2_ with membrane sites for the stimulation of signal transduction pathways has not yet been demonstrated, a study revealed for the first time the involvement of the TLRs in the inflammatory response induced by MT-III (Asp49-PLA_2_) from *B. asper* in macrophages [205]. The involvement of TLR2 and MyD88 adapter molecules was demonstrated to be critical in producing PGs, COX-2 protein expression, and cytokines IL-1β and IL-10 induced by this svPLA_2_. An indirect mechanism for the activation of TLRs through the release of DAMPs was suggested by the authors, since the analysis of the fatty acids released by the hydrolysis of membrane phospholipids by MT-III revealed high levels of oleic and palmitic acids. In this context, it is known that arachidonic, oleic, and palmitic acids produced by membrane cleavage by sPLA_2_s are important bioactive mediators involved in the induction and release of COX-2 and PGE_2_, respectively, through the activation of intracellular signaling mechanisms in several cell types [206,207,208,209].

### 2.3. Bothrops svPLA_2s_ Trigger Lipid Accumulation in Immunocompetent Cells

As mentioned, the high enzymatic activity exhibited by ophidian sPLA_2_ provides a microenvironment rich in free fatty acids that exert stimulating effects in immunocompetent cells, leading to the biosynthesis of lipid mediators. Moreover, excess free fatty acids exert cytotoxic effects that trigger the activation of cellular mechanisms capable of converting free fatty acids into metabolites of lower toxicity, known as neutral lipids (triacylglycerol and cholesterol—energetic body reserve) [210]. In recent decades, numerous studies have demonstrated the activation of intracellular metabolic pathways responsible for the metabolization of free fatty acids in neutral lipids and the consequent formation of dynamic organelles called lipid droplets (LDs) [211,212]. These organelles are composed of a hydrophobic neutral lipid core surrounded by a phospholipid monolayer membrane, which contains numerous proteins related to cellular activation in addition to structural proteins, such as perilipin 2 (PLIN2), which plays an important role in LD assembly and the formation of foam macrophage [213], marker cells in metabolic diseases, such as atherosclerosis and obesity [214]. LDs are commonly present in adipocytes due to the already-established role of these cells in the supply of energy in mammalian organisms [215]. In addition to the relevance regarding lipid homeostasis, the direct relationship between increased LD formation and inflammatory processes was evidenced by numerous studies [216,217,218]. In this sense, mammalian group IIA sPLA_2_s have been identified as potential plasma biomarkers for diseases related to lipid imbalance, such as atherosclerotic cardiovascular disease and obesity [219]. Alongside a marked inflammatory reaction, these metabolic diseases are characterized by lipid accumulation in immunocompetent cells [220,221]. In line with the ability of svPLA_2_s to elicit an inflammatory response characterized by a high level of inflammatory mediators and free fatty acids, it was demonstrated that MT-III (Asp49 PLA_2_), isolated from *B. asper* venom, induced LD formation enriched by PLIN2 protein in mice peritoneal macrophages [170]. This effect was likewise observed in rat vascular smooth muscle cells isolated from the thoracic aorta stimulated by MT-III [168]. Moreover, the ability of MT-II, a Lys49 PLA_2_ homologue devoid of catalytic activity from *B. asper* venom, to directly activate macrophages to form LDs was reported [167]. This effect was reproduced by a synthetic peptide corresponding to the C-terminal sequence 115–129 of MT-II, evidencing the critical role of C-terminus for the MT-II-induced effect [167]. Similarly, BaTX-I, a catalytically inactive Lys49 variant; BaTX-II, a catalytically active Asp49; and BaPLA_2_, an acidic catalytically active Asp49 PLA_2_, isolated from *B. atrox* snake venom, increased the number of LDs on murine macrophages cell line J774A.1 [152]. The formation of LDs upon stimulus by MT-III was likewise demonstrated in human monocytes of peripheral blood [222].

Considering the above information, LD formation induced by svPLA_2_s in phagocytes proved to be inherent to the action of sPLA_2_s regardless of the catalytic activity. The capability of *Bothrops* svPLA_2_s to induce the formation of LDs is related to the activation of PRRs of an innate immune response, kinase proteins, and intracellular PLA_2_ signaling pathways involved in cellular metabolism, proliferation, and differentiation [170,205]. Hence, by using gene knockout mice cells and a pharmacological approach, the participation of TLR2, MYD 88 adaptor protein, and CD36 in LD formation in macrophages in culture has been reported [170,205]. In addition, the upregulation of CD36 receptors was observed in these cells. Considering the participation of the MYD 88 adaptor molecule, the involvement of other TLRs has yet to be investigated. In line with the reported Asp49 svPLA_2_ action on PRRs, the upregulation of both SRA-1, from the scavenger receptor family, and LOX-1, an LDL receptor, was demonstrated in mouse aortic smooth muscle cells (VSMCs) [168]. These findings demonstrate that the inflammatory response elicited by Asp49 svPLA_2_ likewise involved the upregulation of PRRs associated with lipid uptake in immunocompetent cells. This fact indicates that svPLA_2_s can be useful tools in studies aiming to understand the diseases associated with lipid imbalance.

In addition to providing the synthesis of mediators, TLR2 activation elicited by MT-III action was related to cytoskeleton activation [205], a critical step in the transport of structural proteins into LDs, such as PLIN2. Cytoskeletal activation involves the activation of kinase proteins [223]. Consistent with this information, the participation of kinase proteins in LD formation induced by both Asp49 and Lys49 svPLA_2_s from *B. asper* venom has been demonstrated through a pharmacological approach and the detection of phosphorylated kinase proteins. Yet, MT-II- and MT-III-induced LD biogenesis is dependent on the activation of PKC, PI3K, p38MAPK, and ERK1/2 signaling pathways in mice peritoneal macrophages [224]. It is well known that PKC regulates a variety of processes associated with lipid droplet biology, such as adipocyte differentiation [225], magnolol-induced lipolysis [226], cholesterol-induced targeting of caveolin to lipid droplets [227], and the expression of the PAT family [228]. Hence, the activation of the PKC signaling pathway in macrophages stimulated by the *Bothrops* PLA_2_s MT-II and MT-III in peritoneal macrophages may be implicated in an increase in PLIN2 protein expression, since LD formation induced by both svPLA_2_s has always been accompanied by an increase in PLIN2 protein expression in macrophages.

PI3K/AKT is a classical pathway involved in insulin resistance, cell growth, and lipid metabolism associated with the inhibition of cholesterol efflux leading to LD formation [229]. In this sense, the participation of the PIK3 signaling pathway in MT-III-induced LDs in macrophages and vascular smooth muscle cells stimulated with MT-III has been demonstrated [167,224]. In the case of vascular smooth muscle cells, the activation of PI3K was related to the uptake of fatty acids to LDs by macropinocytosis [167]. Furthermore, the findings that MT-III increases phagocytic activity and upregulates macrophage markers in VSMCs reinforce the importance of this class of enzymes as inducers of factors implicated in the formation of foamy cells in both mononuclear phagocytic cells and VSMCs, which are key elements in the development of metabolic diseases.

The MAPK signaling pathway has been revealed to mediate the activation of intracellular PLA_2_s in physiologic and inflammatory contexts [230,231]. It has been demonstrated that the ERK1/2 signaling pathway is implicated in LD formation via the activation of phospholipase D (PLD) and phosphorylation of dynein [232,233]. Consistent with this information, the critical role of ERK1/2 for LD formation induced by MT-II and MT-III in macrophages and VSMCs was reported [167,224]. Regarding p38 MAPK, the literature evidenced its importance in the development of atherosclerosis [234] and the apoptosis of foam macrophages. Macrophage death is a feature of atherosclerotic plaque linked to necrosis and plaque destabilization [235]. Interestingly, although MT-III has been demonstrated to activate apoptotic pathways, including the p38 MAPK signaling pathway, DGAT, ACAT, cPLA_2_, and LD formation in macrophages, no change in cell viability was observed. Further studies may clarify this lack of apoptotic effect.

It is known that the MAP kinases signaling pathway is implicated in intracellular PLA_2_ activation, including the Ca^2+^-dependent cytosolic group IVA PLA_2_ (cPLA_2α_) and the Ca^2+^-independent group VIA PLA_2_ (iPLA_2_) involved in both physiological and pathophysiological conditions [236,237]. The biogenesis of LDs in CHO-K1 cells submitted to an enriched environment of fatty acids demonstrated ERK, p38, and JNK signaling pathway activation, with JNK cascade being responsible for cPLA_2α_ phosphorylation in this event. Of note, cPLA_2_ was likewise implicated in LD biogenesis stimulated by MT-III associated with the activation of ERK and the p38 MAPK signaling pathway [224]. Considering the ability of cPLA_2_ to mobilize cell membrane fatty acids [238], the aforementioned activation should amplify the action of MT-III and provide a greater substrate for the metabolization and formation of LDs. Moreover, the biogenesis of LDs induced by the svPLA_2_s MT-III and MT-II is dependent on the activation of iPLA_2_ signaling pathways [167,224]. This signaling pathway was associated with the processing of fatty acids into triacylglycerol, a relevant component in the constitution of LDs [214]. Hence, the crosstalk already evidenced between intracellular PLA_2_ and svPLA_2_ to elicit inflammatory conditions [169] might contribute to elucidating mechanisms related to the formation of LDs.

**Figure 4:**
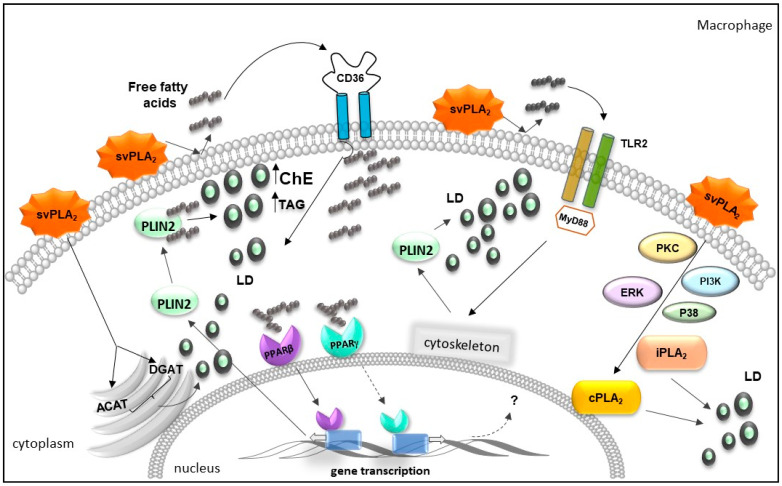
Schematic representation of the mechanisms and factors involved in lipid accumulation induced by svPLA_2_s, in macrophages. svPLA_2_ acts on membrane phospholipids generating free fatty acids, which are ligands of and may activate the TLR2, CD36, and cytoplasmic transcription receptors and factors PPARs. svPLA_2_ induces the activation of transcription factors PPAR-γ and PPAR-δ/β and increases protein expression of PPARs and CD36. PPAR-γ, PPAR-β/δ, TLR2/MyD88, and CD36 receptors, as well as DGAT and ACAT enzymes are involved in the lipid droplets formation stimulated by MT-III. PPAR-β/δ, but not PPAR-γ, is implicated in upregulation of PLIN2 protein expression, induced by MT-III. Moreover, TLR2 and the Myd88 adaptor molecules participate in the recruitment of the PLIN2 protein via cytoskeleton activation stimulated by MT-III. In addition, LD formation induced by svPLA_2_ is dependent on activation of PKC, PI3K, p38MAP, ERK, cPLA_2_, and iPLA_2_ signaling pathways.

The peroxisome proliferator-activated receptors (PPARs) are transcription factors belonging to the family of nuclear receptors that regulate glucose homeostasis, inflammation, and lipid metabolism. Three proteins, encoded by distinct genes, have been identified: PPAR-α, PPAR-β/δ, and PPAR-γ, which control gene expression by binding to PPREs in the promoters [239]. The activation of PPARs is a tightly regulated process implicated in the control of lipid homeostasis, which involves the biogenesis of LDs and protein expression involved in lipid uptake, including PRRs and structural protein PLIN2, and enzymes implicated in neutral lipid synthesis (triacylglycerol and cholesterol) [240]. PPARs have been demonstrated to increase in foam macrophages [241]. In this sense, it was revealed that MT-III induced the upregulation of the transcription factors PPAR-γ and PPAR-β/δ, in addition to the translocation of these factors to the nucleus of mouse peritoneal macrophages. The pharmacological blockage of the PPAR-β/δ transcription factor abolished the increase in PLIN2 and CD36 protein expression induced by MT-III. Moreover, the PPAR-γ blockage caused a reduction in LD formation and abolished CD36 receptor protein expression induced by MT-III. Since an increased expression of CD36 and PLIN2 is related to macrophage differentiation into foam cells [242,243], these findings suggest that MT-III induces foam cell formation by this route. In addition, MT-III caused an increase in the levels of triacylglycerol and cholesterol due to the uptake of free fatty acids. These effects were mediated by DGAT and ACAT enzymes, which are involved in the synthesis of triacylglycerol and cholesterol, respectively [244]. In agreement with this study, a significant increase in triacylglycerol and cholesterol levels was observed in human monocytes under MT-III stimulation [222]. This effect was dependent on fatty acid reacylation. Moreover, the fatty acid composition of triacylglycerol and cholesterol induced by MT-III was compatible with fatty acids released by the enzymatic action of this svPLA_2_ on cell membranes. According to the above information, the mechanisms triggered by MT-III, in both mice peritoneal macrophages and human monocytes, align with macrophages differentiation into foamy cells, a cell type characteristic of inflammatory metabolic diseases, such as atherosclerosis [245]. Similarly, MT-III could stimulate LD formation in VSCMs. This lipid accumulation was likewise mediated by the activation of transcription factors PPAR-γ and PPAR-β/δ and DGAT and ACAT enzymes. Moreover, it is noteworthy that VSCMs under stimulation of MT-III exhibited an increase in the protein levels of PRRs, SRA-1 (scavenger receptor type 1), and LOX-1 (lectin-like oxidized low-density lipoprotein receptor-1). Interestingly, the blockage of these receptors did not alter the formation of LDs induced by MT-III, but the upregulation of LOX-1 was associated with an increased uptake of acetylated-low density lipoprotein (acLDL) in VSMCs stimulated by this svPLA_2_. This higher uptake of acLDL by VSMCs identifies new pathways involved in the accumulation of lipids triggered by a sPLA_2_ that is not directly linked to the reacylation of free fatty acids. In addition, lipid accumulation induced by MT-III in VSCMs was related to the expression of ATP-binding cassette transporters ABCA1 and ABCG1, responsible for the efflux of cholesterol of macrophage-derived foam cells [242]. Although the signaling pathway by which MT-III induces an increased expression of the factors implicated in lipid homeostasis has not been fully elucidated, these studies have broadened the knowledge about the actions of svPLA_2_s on the formation of LDs and the synthesis of lipid mediators and provided new insights into the actions of group IIA sPLA_2_s in diseases related to lipid imbalance. The pathways and factors involved in lipid accumulation in an innate immune cell (macrophage) upon stimulus by svPLA_2_s are summarized in Figure 4.

Another aspect related to the metabolism of free fatty acids is the biosynthesis of lipid mediators [246,247,248]. On the one hand, it is known that the synthesis of eicosanoids is closely related to the triggering of the inflammatory process induced by svPLA_2_s, regardless of the catalytic activity on the cell membranes [13]. In this sense, the immunofluorescence approach has demonstrated that LDs stimulated by svPLA_2_s synthesize PGE_2_ [167,249]. On the other hand, some eicosanoids are implicated in the resolution of inflammatory processes, such as PGJ_2_. This mediator was co-located in LDs in macrophage peritoneal mice stimulated by MT-II [161], indicating, for the first time, that the LDs not only are related to the production of inflammatory mediators but also might play a role in regulating this process.

Adipose tissue is the principal organ responsible for balancing energy metabolism in the mammalian body. An imbalance in adipose tissue functions is linked to the triggering of the inflammatory process observed in metabolic diseases, including obesity [250,251]. Recently, it has been demonstrated that MT-III activated proinflammatory mechanisms in 3T3-L1 preadipocytes, including the biosynthesis of PGE_2_ and PGI_2_, lipid mediators implicated in preadipocytes differentiation into mature adipocytes, and IL-6 and MCP-1 [169]. In these cells, PGE_2_ production induced by MT-III was dependent on cPLA_2_ activation, the upregulation of COX-2 and mPGES-1, and the engagement of the PGE_2_ EP4 receptor. In addition, the release of IL-6 and MCP-1 was dependent on EP4 or EP3 activation, respectively. These data indicate that the production of PGE_2_ is critical for the activation of proinflammatory pathways associated with cytokine production in preadipocytes stimulated by this svPLA_2_. Furthermore, MT-III upregulated the gene expression of the adipokines leptin and adiponectin in preadipocytes [169]. These mediators have been described as regulating appetite and satiety, glucose and lipid metabolism, inflammation, and immune functions [252,253,254,255]. Although the mechanisms related to the release of adipokines and the ability of MT-III to induce lipid accumulation in adipocytes have not yet been investigated, these data offer new directions for investigating the actions triggered by svPLA_2_s and mammalian GIIA sPLA_2_s.

## 3. Conclusions

The existing literature demonstrates that the svPLA_2_s trigger a cascade of inflammatory events including edema formation, leukocyte recruitment into tissues, release of a complex network of inflammatory mediators, and increased oxidative stress in experimental animal models that mimic the inflammatory responses elicited by viperid snake venoms, especially those from the *Bothrops* genus, in the victims. The catalytic activity of the svPLA_2_s is not strictly required by these proteins for the triggering of all the inflammatory responses, since the catalytically inactive Lys49 PLA_2_ variants can display inflammatory events that are qualitatively similar to those of Asp49 PLA_2_s. In addition to cell migration, the svPLA_2_s can activate distinct functions of immunocompetent cells that include phagocytosis, the respiratory burst, NET formation, production of cytokines, chemokines and multiple reactive cleavage products such as lysophospholipids, polyunsaturated fatty acids and eicosanoids, as well as formation of LDs. The highly complex network of mediators, particularly lipid mediators, modulates a variety of inflammatory events triggered by this class of snake venom toxins. The effects triggered by svPLA_2_s in inflammatory cells that lead to generation of lipid mediators has been associated with the activation of distinct signaling pathways of inflammatory kinase proteins by mechanisms dependent and independent of NF-kB. Moreover, the inflammatory response elicited by svPLA_2_s in leukocytes also involves upregulation of PRRs of innate immune response, the crosstalk between the svPLA_2_ and intracellular PLA_2_s, and upregulation of factors implicated in lipid homeostasis. Although much has been learned regarding the inflammatory actions of svPLA_2_s, many knowledge gaps still exist and need to be addressed. There is still considerable work to be done before we fully understand the complex interactions that occur among svPLA_2_s and immunocompetent cells and tissues that lead to inflammation. The cell acceptors and/or receptors involved in the actions of svPLA_2_s in these cells and the signaling pathways elicited and how they interact with each other remain to be clarified. In addition, the actual types and subtypes of receptors activated by the principal mediators produced by svPLA_2_s and the mechanisms involved in coupling between the svPLA_2_s and endogenous PLA_2_s have yet to be investigated. Recently, the stimulatory activity of a svPLA_2_ on adipose tissue cells leading to increased biosynthesis of PGE_2_ and other inflammatory mediators including adipokines was demonstrated. This information offers new directions for investigating the actions triggered by svPLA_2_s and mammalian GIIA sPLA_2_ and gives insights into the potential role of the adipocytes as target cells for viperid snake venoms. Finally, a deeper and comprehensive understanding of the mechanisms underlying the inflammatory actions of svPLA_2_s will give new insights into (i) the actions of group IIA sPLA_2_s in diseases related to lipid imbalance and inflammation and (ii) a better understanding of the pathophysiology of *Bothrops* envenomation. Within this frame, the acquired knowledge might pave the way for the development of novel therapeutic approaches aimed at counteracting the prominent inflammation caused by *Bothrops* snakebite envenoming.

## Figures and Tables

**Table 1:** Inflammatory activities of basic and acidic svPLA_2_s isoforms.

PLA_2_	Origin	Basic or Acid	Type of PLA_2_ Variant	Inflammatory Activity/ Experimental Model	Refs.
Piratoxin-I	*B. pirajai*	Basic	Lys49	Increase in vascular permeability (in vivo) Mast cell degranulation, neutrophil chemotaxis (in vitro)	[125,136]
P-1	*B. neuwiedii*	Acidic	nd	Edema (in vivo)	[126]
P-2	*B. neuwiedii*	Acidic	nd	Edema (in vivo)	[126]
SIIISPIIA	*B. jararacussu*	Acidic	Asp49	Edema (in vivo)	[129]
SIIISPIIB	*B. jararacussu*	Acidic	Asp49	Edema (in vivo)	[129]
SIIISPIIIA	*B. jararacussu*	Acidic	Asp49	Edema (in vivo)	[129]
SIIISPIIIB	*B. jararacussu*	Acidic	Asp49	Edema (in vivo)	[129]
BintTX-I	*B. insularis*	Acidic	Asp49	Edema (in vivo)	[130]
Bothropstoxin-I (BthTX-I)	*B. jararacussu*	Basic	Lys49	Edema, leukocyte migration, mast cell degranulation (in vivo) Neutrophil chemotaxis, activation of inflammasome (in vitro)	[128,131,136,147,148]
Bothropstoxin-II (BthTX-II)	*B. jararacussu*	Basic	Asp49	Edema, leukocyte migration, mast cell degranulation (in vivo) Neutrophil chemotaxis (in vitro)	[128,131,136]
Myotoxin-II (MT-II)	*B. asper*	Basic	Lys49	Increase in vascular permeability, leukocyte migration, release of mediators, hyperalgesia, eicosanoid production, COX-2 expression (in vivo) Phagocytosis, H_2_O_2_ production COX-2 expression, lipid droplet formation (in vitro)	[123,126,135,138,161,162,163,164,165,166,167]
Myotoxin-III (MT-III)	*B. asper*	Basic	Asp49	Increase in vascular permeability, leukocyte migration, release of mediators, hyperalgesia, eicosanoid production; COX-2 expression (in vivo) Phagocytosis, H_2_O_2_ production, COX-2 expression, lipid droplet formation, preadipocyte activation (in vitro)	[123,126,135,138,162,163,164,165,166,168,169,170]
BnSP-7	*B. pauloensis*	Basic	Lys49	Edema (in vivo)	[139]
BatroxPLA_2_	*B. atrox*	Acidic	Asp49	Leukocyte chemotaxis, mediators release (in vivo) Mast cell degranulation (in vitro)	[140]
BJ-PLA_2_-I	*B. jararaca*	Acidic	Asp49	Leukocyte migration, mediators release (in vivo)	[141]
Bbil-TX	*B. bilineata*	Basic	nd	Neutrophil migration, mediators release (in vivo)	[145]
BmaTX-I	*B. mattogrossensis*	Basic	Lys49	Mediator release (in vitro)	[146]
BmaTX-II	*B. mattogrossensis*	Basic	Lys49	Mediator release (in vitro)	[146]
BaltTX-I	*B. alternatus*	Basic	Lys49	Phagocytosis, superoxide production (in vitro)	[149]
BaltTX-II	*B. alternatus*	Basic	Asp49	Superoxide production (in vitro)	[149]
pMTX-II	*B. asper*	Basic	Lys49	Phagocytosis, superoxide production (in vitro)	[150]
pMTX-III	*B. asper*	Basic	Asp49	Phagocytosis, superoxide production (in vitro)	[150]
pMTX-IV	*B. asper*	Basic	Lys49	Phagocytosis, superoxide production (in vitro)	[150]
BaTX-I	*B. atrox*	Basic	Lys49	Superoxide production, lipid droplet formation (in vitro)	[155]
BaTX-II	*B. atrox*	Basic	Asp49	Superoxide and H_2_O_2_ production, MPO release, NET formation, lipid droplet formation (in vitro)	[155]
BaPLA_2_	*B. atrox*	Acidic	Asp49	Superoxide production, lipid droplet formation (in vitro)	[140,155]
BaPLA_2_I	*B. atrox*	Basic	nd	Mast cell degranulation, edema (in vivo)	[132]
BaPLA_2_III	*B. atrox*	Neutral	nd	Mast cell Degranulation, edema (in vivo)	[132]

nd, not described.

## Data Availability

Not applicable.

## References

[1] Teixeira C.F.P., Cury Y., Oga S., Jancar S. (1994). Hyperalgesia induced by *Bothrops jararaca* venom in rats: Role of eicosanoids and platelet activating factor (PAF). Toxicon.

[2] Teixeira C., Cury Y., Moreira V., Picolo G., Chaves F. (2009). Inflammation induced by *Bothrops asper* venom. Toxicon.

[3] Mamede C.C.N., de Sousa Simamoto B.B., da Cunha Pereira D.F., de Oliveira Costa J., Ribeiro M.S.M., de Oliveira F. (2020). Edema, hyperalgesia and myonecrosis induced by Brazilian bothropic venoms: Overview of the last decade. Toxicon.

[4] Gutiérrez J.M., Rucavado A., Chaves F., Díaz C., Escalante T. (2009). Experimental pathology of local tissue damage induced by *Bothrops asper* snake venom. Toxicon.

[5] (2019). Ministério da Saúde Guia de Vigilância Epidemiológica.

[6] Echeverría S., Leiguez E., Guijas C., do Nascimento N.G., Acosta O., Teixeira C., Leiva L.C., Rodríguez J.P. (2018). Evaluation of pro-inflammatory events induced by Bothrops alternatus snake venom. Chem. Biol. Interact..

[7] Jorge R.J.B., Monteiro H.S.A., Gonçalves-Machado L., Guarnieri M.C., Ximenes R.M., Borges-Nojosa D.M., Luna K.P.D.O., Zingali R.B., Corrêa-Netto C., Gutiérrez J.M. (2015). Venomics and antivenomics of *Bothrops erythromelas* from five geographic populations within the *Caatinga ecoregion* of northeastern Brazil. J. Proteom..

[8] Nicolau C.A., Carvalho P.C., Junqueira-de-Azevedo I.L.M., Teixeira-Ferreira A., Junqueira M., Perales J., Neves-Ferreira A.G.C., Valente R.H. (2017). An in-depth snake venom proteopeptidome characterization: Benchmarking *Bothrops jararaca*. J. Proteom..

[9] Sousa L.F., Portes-Junior J.A., Nicolau C.A., Bernardoni J.L., Nishiyama M.Y., Amazonas D.R., Freitas-de-Sousa L.A., Mourão R.H., Chalkidis H.M., Valente R.H. (2017). Functional proteomic analyses of Bothrops atrox venom reveals phenotypes associated with habitat variation in the Amazon. J. Proteom..

[10] De Farias I.B., de Morais-Zani K., Serino-Silva C., Sant’Anna S.S., da Rocha M.M., Grego K.F., Andrade-Silva D., Serrano S.M.T., Tanaka-Azevedo A.M. (2018). Functional and proteomic comparison of *Bothrops jararaca* venom from captive specimens and the Brazilian Bothropic Reference Venom. J. Proteom..

[11] Mora-Obando D., Salazar-Valenzuela D., Pla D., Lomonte B., Guerrero-Vargas J.A., Ayerbe S., Gibbs H.L., Calvete J.J. (2020). Venom variation in *Bothrops asper* lineages from North-Western South America. J. Proteom..

[12] Gutiérrez J.M., Lomonte B. (2013). Phospholipases A2: Unveiling the secrets of a functionally versatile group of snake venom toxins. Toxicon.

[13] Bickler P.E. (2020). Amplification of Snake Venom Toxicity by Endogenous Signaling Pathways. Toxins.

[14] Hiu J.J., Yap M.K.K. (2020). Cytotoxicity of snake venom enzymatic toxins: Phospholipase A2 and L-amino acid oxidase. Biochem. Soc. Trans..

[15] Gutiérrez J.M., Chaves F., Bolaños R., Cerdas L., Rojas E., Arroyo O., Portilla E. (1981). Neutralizacion de los efectos locales del veneno de *Bothrops asper* por un antiveneno polivalente. Toxicon.

[16] Gutiérrez J.M., Rojas G., Bogarín G., Lomonte B. (1996). Evaluation of the neutralizing ability of antivenoms for the treatment of snake bite envenoming in Central America. Envenomings Treat..

[17] Gutiérrez J., Avila C., Rojas E., Cerdas L. (1988). An alternative in vitro method for testing the potency of the polyvalent antivenom produced in Costa Rica. Toxicon.

[18] Cardoso J.L., Fan H.W., França F.O., Jorge M.T., Leite R.P., Nishioka S.A., Avila A., Sano-Martins I.S., Tomy S.C., Santoro M.L. (1993). Randomized comparative trial of three antivenoms in the treatment of envenoming by lance-headed vipers (*Bothrops jararaca*) in São Paulo, Brazil. Q. J. Med..

[19] Lewin M., Samuel S., Merkel J., Bickler P. (2016). Varespladib (LY315920) appears to be a potent, broad-spectrum, inhibitor of snake venom phospholipase A2 and a possible pre-referral treatment for envenomation. Toxins.

[20] Wang Y., Zhang J., Zhang D., Xiao H., Xiong S., Huang C. (2018). Exploration of the inhibitory potential of varespladib for snakebite envenomation. Molecules.

[21] Schaloske R.H., Dennis E.A. (2006). The phospholipase A2 superfamily and its group numbering system. Biochim. Biophys. Acta-Mol. Cell Biol. Lipids.

[22] Six D.A., Dennis E.A. (2000). The expanding superfamily of phospholipase A2 enzymes: Classification and characterization. Biochim. Biophys. Acta-Mol. Cell Biol. Lipids.

[23] Dennis E.A., Cao J., Hsu Y.H., Magrioti V., Kokotos G. (2011). Phospholipase A2 enzymes: Physical structure, biological function, disease implication, chemical inhibition, and therapeutic intervention. Chem. Rev..

[24] Murakami M., Sato H., Taketomi Y. (2020). Updating Phospholipase A2 Biology. Biomolecules.

[25] Lee G.-H., Fujita M., Nakanishi H., Miyata H., Ikawa M., Maeda Y., Murakami Y., Kinoshita T. (2020). PGAP6, a GPI-specific phospholipase A2, has narrow substrate specificity against GPI-anchored proteins. J. Biol. Chem..

[26] Aloulou A., Rahier R., Arhab Y., Noiriel A., Abousalham A., Sandoval G. (2018). Phospholipases: An Overview. Methods in Molecular Biology.

[27] Quach N.D., Arnold R.D., Cummings B.S. (2014). Secretory phospholipase A2 enzymes as pharmacological targets for treatment of disease. Biochem. Pharmacol..

[28] Harris J.B., Scott-Davey T. (2013). Secreted phospholipases A2 of snake venoms: Effects on the peripheral neuromuscular system with comments on the role of phospholipases A2 in disorders of the CNS and their uses in industry. Toxins.

[29] Triggiani M., Granata F., Frattini A., Marone G. (2006). Activation of human inflammatory cells by secreted phospholipases A2. Biochim. Biophys. Acta Mol. Cell Biol. Lipids.

[30] Valentin E., Lambeau G. (2000). What can venom phospholipases A2 tell us about the functional diversity of mammalian secreted phospholipases A2?. Biochimie.

[31] Kudo I., Murakami M. (2002). Phospholipase A2 enzymes. Prostaglandins Other Lipid Mediat..

[32] Kini R.M. (2003). Excitement ahead: Structure, function and mechanism of snake venom phospholipase A2 enzymes. Toxicon.

[33] Lambeau G., Barhanin J., Schweitz H., Qar J., Lazdunski M. (1989). Identification and properties of very high affinity brain membrane-binding sites for a neurotoxic phospholipase from the taipan venom. J. Biol. Chem..

[34] Lambeau G., Schmid-Alliana A., Lazdunski M., Barhanin J. (1990). Identification and purification of a very high affinity binding protein for toxic phospholipases A2 in skeletal muscle. J. Biol. Chem..

[35] Silliman C.C., Moore E.E., Zallen G., Gonzalez R., Johnson J.L., Elzi D.J., Meng X., Hanasaki K., Ishizaki J., Arita H. (2002). Presence of the M-type sPLA 2 receptor on neutrophils and its role in elastase release and adhesion. Am. J. Physiol. Physiol..

[36] Granata F., Petraroli A., Boilard E., Bezzine S., Bollinger J., Del Vecchio L., Gelb M.H., Lambeau G., Marone G., Triggiani M. (2005). Activation of Cytokine Production by Secreted Phospholipase A 2 in Human Lung Macrophages Expressing the M-Type Receptor. J. Immunol..

[37] Hanasaki K., Arita H. (2002). Phospholipase A2 receptor: A regulator of biological functions of secretory phospholipase A2. Prostaglandins Other Lipid Mediat..

[38] Ezekowitz R.A.B., Stahl P.D. (1988). The structure and function of vertebrate mannose lectin-like proteins. J. Cell Sci..

[39] Taylor M.E., Conary J.T., Lennartz M.R., Stahl P.D., Drickamer K. (1990). Primary structure of the mannose receptor contains multiple motifs resembling carbohydrate-recognition domains. J. Biol. Chem..

[40] Gantzel R.H., Kjær M.B., Laursen T.L., Kazankov K., George J., Møller H.J., Grønbæk H. (2021). Macrophage Activation Markers, Soluble CD163 and Mannose Receptor, in Liver Fibrosis. Front. Med..

[41] Gordon S., Clarke S., Greaves D., Doyle A. (1995). Molecular immunobiology of macrophages: Recent progress. Curr. Opin. Immunol..

[42] Stoy N. (2001). Macrophage Biology and Pathobiology in the Evolution of Immune Responses: A Functional Analysis. Pathobiology.

[43] Hernández M., Burillo S.L., Crespo M.S., Nieto M.L. (1998). Secretory Phospholipase A2 Activates the Cascade of Mitogen-activated Protein Kinases and Cytosolic Phospholipase A2 in the Human Astrocytoma Cell Line 1321N1. J. Biol. Chem..

[44] Kinoshita E., Handa N., Hanada K., Kajiyama G., Sugiyama M. (1997). Activation of MAP kinase cascade induced by human pancreatic phospholipase A2 in a human pancreatic cancer cell line. FEBS Lett..

[45] Kundu G.C., Mukherjee A.B. (1997). Evidence that porcine pancreatic phospholipase A2 via its high affinity receptor stimulates extracellular matrix invasion by normal and cancer cells. J. Biol. Chem..

[46] Triggiani M., Calabrese C., Granata F., Gentile M., Marone G. (2000). Metabolism of Lipid Mediators in Human Eosinophils. Human Eosinophils.

[47] Hara S., Kudo I., Matsuta K., Miyamoto T., Inoue K. (1988). Amino Acid Composition and NH2-Terminal Amino Acid Sequence of Human Phospholipase A2 Purified from Rheumatoid Synovial Fluid1. J. Biochem..

[48] Touqui L., Alaoui-El-Azher M. (2001). Mammalian Secreted Phospholipases A2 and Their Pathophysiolo-gical Significance in Inflammatory Diseases. Curr. Mol. Med..

[49] Liu N.J., Chapman R., Lin Y., Mmesi J., Bentham A., Tyreman M., Abraham S., Stevens M.M. (2016). Point of care testing of phospholipase A2 group IIA for serological diagnosis of rheumatoid arthritis. Nanoscale.

[50] Feldman M., Ginsburg I. (2021). A Novel Hypothetical Approach to Explain the Mechanisms of Pathogenicity of Rheumatic Arthritis. Mediterr. J. Rheumatol..

[51] Aufenanger J., Samman M., Quintel M., Fassbender K., Zimmer W., Bertsch T. (2002). Pancreatic phospholipase A2 activity in acute pancreatitis: A prognostic marker for early identification of patients at risk. Clin. Chem. Lab. Med..

[52] Zhang K.J., Zhang D.L., Jiao X.L., Dong C. (2013). Effect of phospholipase A2 silencing on acute experimental pancreatitis. Eur. Rev. Med. Pharmacol. Sci..

[53] Schröder T., Kivilaakso E., Kinnunen P.K.J., Lempinen M. (1980). Serum Phospholipase A 2 in Human Acute Pancreatitis. Scand. J. Gastroenterol..

[54] Vadas P., Pruzanski W. (1986). Role of secretory phospholipases A2 in the pathobiology of disease. Lab. Investig..

[55] Ahmad N.S., Tan T.L., Arifin K.T., Ngah W.Z.W., Yusof Y.A.M. (2020). High sPLA2-IIA level is associated with eicosanoid metabolism in patients with bacterial sepsis syndrome. PLoS ONE.

[56] Haapamäki M.M., Grönroos J.M., Nurmi H., Irjala K., Alanen K.A., Nevalainen T.J. (1999). Phospholipase A2 in serum and colonic mucosa in ulcerative colitis. Scand. J. Clin. Lab. Investig..

[57] Haapamäki M.M., Grönroos J.M., Nurmi H., Alanen K., Nevalainen T.J. (1999). Gene Expression of Group Ii Phospholipase A2 in Intestine in Crohn’s Disease. Am. J. Gastroenterol..

[58] Woodruff T.M., Arumugam T.V., Shiels I.A., Newman M.L., Ross P.A., Reid R.C., Fairlie D.P., Taylor S.M. (2005). A potent and selective inhibitor of group IIa secretory phospholipase A2 protects rats from TNBS-induced colitis. Int. Immunopharmacol..

[59] Stadel J.M., Hoyle K., Naclerio R.M., Roshak A., Chilton F.H. (1994). Characterization of phospholipase A2 from human nasal lavage. Am. J. Respir. Cell Mol. Biol..

[60] Kim D.K., Fukuda T., Thompson B.T., Cockrill B., Hales C., Bonventre J.V. (1995). Bronchoalveolar lavage fluid phospholipase A2 activities are increased in human adult respiratory distress syndrome. Am. J. Physiol. Cell. Mol. Physiol..

[61] Letsiou E., Htwe Y.M., Dudek S.M. (2021). Secretory Phospholipase A2 Enzymes in Acute Lung Injury. Cell Biochem. Biophys..

[62] Granata F., Staiano R.I., Loffredo S., Petraroli A., Genovese A., Marone G., Triggiani M. (2010). The role of mast cell-derived secreted phospholipases A2 in respiratory allergy. Biochimie.

[63] Sun C.Q., Zhong C.Y., Sun W.W., Xiao H., Zhu P., Lin Y.Z., Zhang C.L., Gao H., Song Z.Y. (2016). Elevated Type II Secretory Phospholipase A2 Increases the Risk of Early Atherosclerosis in Patients with Newly Diagnosed Metabolic Syndrome. Sci. Rep..

[64] Menschikowski M., Hagelgans A., Siegert G. (2006). Secretory phospholipase A2 of group IIA: Is it an offensive or a defensive player during atherosclerosis and other inflammatory diseases?. Prostaglandins Other Lipid Mediat..

[65] Cunningham T.J., Yao L., Oetinger M., Cort L., Blankenhorn E.P., Greenstein J.I. (2006). Secreted phospholipase A2 activity in experimental autoimmune encephalomyelitis and multiple sclerosis. J. Neuroinflamm..

[66] Mirtti T., Laine V.J.O., Hiekkanen H., Hurme S., Rowe O., Nevalainen T.J., Kallajoki M., Alanen K. (2009). Group IIA phospholipase A2 as a prognostic marker in prostate cancer: Relevance to clinicopathological variables and disease-specific mortality. Apmis.

[67] Dong Z., Liu Y., Scott K.F., Levin L., Gaitonde K., Bracken R.B., Burke B., Zhai Q.J., Wang J., Oleksowicz L. (2010). Secretory phospholipase A2-IIa is involved in prostate cancer progression and may potentially serve as a biomarker for prostate cancer. Carcinogenesis.

[68] Wang M., Hao F.Y., Wang J.G., Xiao W. (2014). Group IIa secretory phospholipase A2 (sPLA2IIa) and progression in patients with lung cancer. Eur. Rev. Med. Pharmacol. Sci..

[69] Chen J., Ye L., Sun Y., Takada Y. (2017). A Concise Update on the Relevance of Secretory Phospholipase A2 Group IIA and its Inhibitors with Cancer. Med. Chem..

[70] Schalkwijk C., Pfeilschifter J., Märki F., van den Bosch H. (1991). Interleukin-1β, tumor necrosis factor and forskolin stimulate the synthesis and secretion of group II phospholipase A2 in rat mesangial cells. Biochem. Biophys. Res. Commun..

[71] Divchev D., Schieffer B. (2008). The secretory phospholipase A2 group IIA: A missing link between inflammation, activated renin-angiotensin system, and atherogenesis?. Vasc. Health Risk Manag..

[72] Leistad L., Feuerherm A.J., Faxvaag A., Johansen B. (2011). Multiple phospholipase A2 enzymes participate in the inflammatory process in osteoarthritic cartilage. Scand. J. Rheumatol..

[73] Beck S., Lambeau G., Scholz-Pedretti K., Gelb M.H., Janssen M.J.W., Edwards S.H., Wilton D.C., Pfeilschifter J., Kaszkin M. (2003). Potentiation of Tumor Necrosis Factor α-induced Secreted Phospholipase A2 (sPLA2)-IIA Expression in Mesangial Cells by an Autocrine Loop Involving sPLA2 and Peroxisome Proliferator-activated Receptor α Activation. J. Biol. Chem..

[74] Wu Y., Li Y., Shang M., Jian Y., Wang C., Bardeesi A.S.A., Li Z., Chen T., Zhao L., Zhou L. (2017). Secreted phospholipase A2 of Clonorchis sinensis activates hepatic stellate cells through a pathway involving JNK signalling. Parasites Vectors.

[75] Sarate R.M., Chovatiya G.L., Ravi V., Khade B., Gupta S., Waghmare S.K. (2016). sPLA 2 -IIA Overexpression in Mice Epidermis Depletes Hair Follicle Stem Cells and Induces Differentiation Mediated Through Enhanced JNK/c-Jun Activation. Stem Cells.

[76] Baek S.-H., Lim J.-H., Park D.-W., Kim S.-Y., Lee Y.-H., Kim J.-R., Kim J.-H. (2001). Group IIA secretory phospholipase A2 stimulates inducible nitric oxide synthase expression via ERK and NF-κB in macrophages. Eur. J. Immunol..

[77] Beck G.C., Yard B.A., Schulte J., Haak M., van Ackern K., van der Woude F.J., Kaszkin M. (2003). Secreted phospholipases A2 induce the expression of chemokines in microvascular endothelium. Biochem. Biophys. Res. Commun..

[78] Medzhitov R. (2008). Origin and physiological roles of inflammation. Nature.

[79] Barton G.M. (2008). A calculated response: Control of inflammation by the innate immune system. J. Clin. Investig..

[80] Kruger P., Saffarzadeh M., Weber A.N.R., Rieber N., Radsak M., von Bernuth H., Benarafa C., Roos D., Skokowa J., Hartl D. (2015). Neutrophils: Between Host Defence, Immune Modulation, and Tissue Injury. PLOS Pathog..

[81] Bonecchi R. (2009). Chemokines and chemokine receptors: An overview. Front. Biosci..

[82] Branco A.C.C.C., Yoshikawa F.S.Y., Pietrobon A.J., Sato M.N. (2018). Role of Histamine in Modulating the Immune Response and Inflammation. Mediat. Inflamm..

[83] Bennett M., Gilroy D.W. (2016). Lipid Mediators in Inflammation. Microbiol. Spectr..

[84] Krieglstein C. (2001). Adhesion molecules and their role in vascular disease. Am. J. Hypertens..

[85] Kameritsch P., Renkawitz J. (2020). Principles of Leukocyte Migration Strategies. Trends Cell Biol..

[86] Rosales C., Uribe-Querol E. (2017). Phagocytosis: A Fundamental Process in Immunity. Biomed Res. Int..

[87] Gordon S. (2016). Phagocytosis: An Immunobiologic Process. Immunity.

[88] Collin M., Ehlers M. (2013). The carbohydrate switch between pathogenic and immunosuppressive antigen-specific antibodies. Exp. Dermatol..

[89] Medzhitov R. (2007). TLR-mediated innate immune recognition. Semin. Immunol..

[90] Aderem A., Ulevitch R.J. (2000). Toll-like receptors in the induction of the innate immune response. Nature.

[91] Kawai T., Akira S. (2007). TLR signaling. Semin. Immunol..

[92] Motta V., Soares F., Sun T., Philpott D.J. (2015). NOD-Like Receptors: Versatile Cytosolic Sentinels. Physiol. Rev..

[93] Corridoni D., Simmons A. (2019). Innate immune receptors for cross-presentation: The expanding role of NLRs. Mol. Immunol..

[94] McGettrick A.F., O’Neill L.A. (2010). Localisation and trafficking of Toll-like receptors: An important mode of regulation. Curr. Opin. Immunol..

[95] Kawai T., Akira S. (2010). The role of pattern-recognition receptors in innate immunity: Update on toll-like receptors. Nat. Immunol..

[96] Broz P., Dixit V.M. (2016). Inflammasomes: Mechanism of assembly, regulation and signalling. Nat. Rev. Immunol..

[97] Rathinam V.A.K., Vanaja S.K., Fitzgerald K.A. (2012). Regulation of inflammasome signaling. Nat. Immunol..

[98] Strowig T., Henao-Mejia J., Elinav E., Flavell R. (2012). Inflammasomes in health and disease. Nature.

[99] Schroder K., Tschopp J. (2010). The Inflammasomes. Cell.

[100] Malik A., Kanneganti T.-D. (2017). Inflammasome activation and assembly at a glance. J. Cell Sci..

[101] Lin Y.-Z., Yao S., Veach R.A., Torgerson T.R., Hawiger J. (1995). Inhibition of Nuclear Translocation of Transcription Factor NF-κB by a Synthetic Peptide Containing a Cell Membrane-permeable Motif and Nuclear Localization Sequence. J. Biol. Chem..

[102] Lawrence T. (2009). The nuclear factor NF-kappaB pathway in inflammation. Cold Spring Harb. Perspect. Biol..

[103] Kwiatkowska K., Sobota A. (1999). Signaling pathways in phagocytosis. Bioessays.

[104] Kitaura J., Eto K., Kinoshita T., Kawakami Y., Leitges M., Lowell C.A., Kawakami T. (2005). Regulation of Highly Cytokinergic IgE-Induced Mast Cell Adhesion by Src, Syk, Tec, and Protein Kinase C Family Kinases. J. Immunol..

[105] Yang Q., Langston J.C., Tang Y., Kiani M.F., Kilpatrick L.E. (2019). The role of tyrosine phosphorylation of protein kinase C delta in infection and inflammation. Int. J. Mol. Sci..

[106] Fruman D.A., Chiu H., Hopkins B.D., Bagrodia S., Cantley L.C., Abraham R.T. (2017). The PI3K Pathway in Human Disease. Cell.

[107] Stephenson J.D., Shepherd V.L. (1987). Purification of the human alveolar macrophage mannose receptor. Biochem. Biophys. Res. Commun..

[108] Ezekowitz R.A., Sastry K., Bailly P., Warner A. (1990). Molecular characterization of the human macrophage mannose receptor: Demonstration of multiple carbohydrate recognition-like domains and phagocytosis of yeasts in Cos-1 cells. J. Exp. Med..

[109] Greenberg S. (1999). Modular components of phagocytosis. J. Leukoc. Biol..

[110] Fernández N., Alonso S., Valera I., Vigo A.G., Renedo M., Barbolla L., Crespo M.S. (2005). Mannose-Containing Molecular Patterns Are Strong Inducers of Cyclooxygenase-2 Expression and Prostaglandin E 2 Production in Human Macrophages. J. Immunol..

[111] McNally A.K., DeFife K.M., Anderson J.M. (1996). Interleukin-4-induced macrophage fusion is prevented by inhibitors of mannose receptor activity. Am. J. Pathol..

[112] Newton R., Holden N. (2003). Inhibitors of p38 Mitogen-Activated Protein Kinase. BioDrugs.

[113] Janssen W.J., Henson P.M. (2012). Cellular Regulation of the Inflammatory Response. Toxicol. Pathol..

[114] Buckley C.D., Gilroy D.W., Serhan C.N. (2014). Proresolving Lipid Mediators and Mechanisms in the Resolution of Acute Inflammation. Immunity.

[115] Serhan C.N., Savill J. (2005). Resolution of inflammation: The beginning programs the end. Nat. Immunol..

[116] Feehan K.T., Gilroy D.W. (2019). Is Resolution the End of Inflammation?. Trends Mol. Med..

[117] Netea M.G., Balkwill F., Chonchol M., Cominelli F., Donath M.Y., Giamarellos-Bourboulis E.J., Golenbock D., Gresnigt M.S., Heneka M.T., Hoffman H.M. (2017). A guiding map for inflammation. Nat. Immunol..

[118] Kini R.M., Chan Y.M. (1999). Accelerated Evolution and Molecular Surface of Venom Phospholipase A2 Enzymes. J. Mol. Evol..

[119] Krizaj I., Bieber A.L., Ritonja A., Gubensek F. (1991). The primary structure of ammodytin L, a myotoxic phospholipase A2 homologue from Vipera ammodytes venom. Eur. J. Biochem..

[120] van den Berg B., Tessari M., de Haas G.H., Verheij H.M., Boelens R., Kaptein R. (1995). Solution structure of porcine pancreatic phospholipase A2. EMBO J..

[121] Díaz C., Gutiérrez J., Lomonte B., Gené J. (1991). The effect of myotoxins isolated from Bothrops snake venoms on multilamellar liposomes: Relationship to phospholipase A2, anticoagulant and myotoxic activities. Biochim. Biophys. Acta-Biomembr..

[122] Lomonte B., Gutiérrez J.M. (2011). Phospholipases A2 from viperidae snake venoms: How do they induce skeletal muscle damage?. Acta Chim. Slov..

[123] Zuliani J.P., Fernandes C.M., Zamuner S.R., Gutiérrez J.M., Teixeira C.F.P. (2005). Inflammatory events induced by Lys-49 and Asp-49 phospholipases A2 isolated from *Bothrops asper* snake venom: Role of catalytic activity. Toxicon.

[124] Teixeira C.F.P., Landucci E.C.T., Antunes E., Chacur M., Cury Y. (2003). Inflammatory effects of snake venom myotoxic phospholipases A2. Toxicon.

[125] Landucci E.C.T., De Castro R.C., Toyama M., Giglio J.R., Marangoni S., De Nucci G., Antunes E. (2000). Inflammatory oedema induced by the Lys-49 phospholipase A2 homologue piratoxin-I in the rat and rabbit. Effect of polyanions and p-bromophenacyl bromide. Biochem. Pharmacol..

[126] Chaves F., León G., Alvarado V.H., Gutiérrez J.M. (1998). Pharmacological modulation of edema induced by Lys-49 and Asp-49 myotoxic phospholipases A2 isolated from the venom of the snake *Bothrops asper* (*Terciopelo*). Toxicon.

[127] Daniele J.J., Bianco I.D., Fidelio G.D. (1995). Kinetic and Pharmacological Characterization of Phospholipases A2 from Bothrops neuwiedii Venom. Arch. Biochem. Biophys..

[128] Landucci E.C., Castro R.C., Pereira M.F., Cintra A.C., Giglio J.R., Marangoni S., Oliveira B., Cirino G., Antunes E., De Nucci G. (1998). Mast cell degranulation induced by two phospholipase A2 homologues: Dissociation between enzymatic and biological activities. Eur. J. Pharmacol..

[129] Ketelhut D.F., Homem de Mello M., Veronese E.L., Esmeraldino L., Murakami M., Arni R., Giglio J., Cintra A.C., Sampaio S. (2003). Isolation, characterization and biological activity of acidic phospholipase A2 isoforms from *Bothrops jararacussu* snake venom. Biochimie.

[130] Cogo J.C., Lilla S., Souza G.H.M.F., Hyslop S., de Nucci G. (2006). Purification, sequencing and structural analysis of two acidic phospholipases A2 from the venom of *Bothrops insularis* (jararaca ilhoa). Biochimie.

[131] de Castro R., Landucci E.C., Toyama M., Giglio J., Marangoni S., De Nucci G., Antunes E. (2000). Leucocyte recruitment induced by type II phospholipases A2 into the rat pleural cavity. Toxicon.

[132] Kanashiro M.M., Rita de Cássia M.E., Petretski J.H., Prates M.V., Alves E.W., Machado O.L., da Silva W.D., Kipnis T.L. (2002). Biochemical and biological properties of phospholipases A2 from *Bothrops atrox* snake venom. Biochem. Pharmacol..

[133] Metcalfe D.D., Baram D., Mekori Y.A. (1997). Mast cells. Physiol. Rev..

[134] Metz M., Maurer M. (2007). Mast cells—Key effector cells in immune responses. Trends Immunol..

[135] Chacur M., Longo I., Picolo G., Gutiérrez J.M., Lomonte B., Guerra J.L., Teixeira C.F.P., Cury Y. (2003). Hyperalgesia induced by Asp49 and Lys49 phospholipases A2 from *Bothrops asper* snake venom: Pharmacological mediation and molecular determinants. Toxicon.

[136] Gambero A., Landucci E.C.T., Toyama M.H., Marangoni S., Giglio J.R., Nader H.B., Dietrich C.P., De Nucci G., Antunes E. (2002). Human neutrophil migration in vitro induced by secretory phospholipases A2: A role for cell surface glycosaminoglycans. Biochem. Pharmacol..

[137] Gambero A., Thomazzi S.M., Cintra A.C.O., Landucci E.C.T., De Nucci G., Antunes E. (2004). Signalling pathways regulating human neutrophil migration induced by secretory phospholipases A2. Toxicon.

[138] Zuliani J.P., Gutiérrez J.M., Teixeira C. (2018). Signaling pathways involved in zymosan phagocytosis induced by two secreted phospholipases A2 isolated from *Bothrops asper* snake venom in macrophages. Int. J. Biol. Macromol..

[139] de Freitas Oliveira C., da Silva Lopes D., Mendes M.M., Homsi-Brandeburgo M.I., Hamaguchi A., de Alcântara T.M., Clissa P.B., de Melo Rodrigues V. (2009). Insights of local tissue damage and regeneration induced by BnSP-7, a myotoxin isolated from Bothrops (neuwiedi) pauloensis snake venom. Toxicon.

[140] Menaldo D.L., Bernardes C.P., Zoccal K.F., Jacob-Ferreira A.L., Costa T.R., Del Lama M.P.F.M., Naal R.M.Z.G., Frantz F.G., Faccioli L.H., Sampaio S.V. (2017). Immune cells and mediators involved in the inflammatory responses induced by a P-I metalloprotease and a phospholipase A2 from Bothrops atrox venom. Mol. Immunol..

[141] Cedro R.C.A., Menaldo D.L., Costa T.R., Zoccal K.F., Sartim M.A., Santos-Filho N.A., Faccioli L.H., Sampaio S.V. (2018). Cytotoxic and inflammatory potential of a phospholipase A2 from *Bothrops jararaca* snake venom. J. Venom. Anim. Toxins Incl. Trop. Dis..

[142] Arend W.P., Gabay C. (2004). Cytokines in the rheumatic diseases. Rheum. Dis. Clin. N. Am..

[143] David B.A., Kubes P. (2019). Exploring the complex role of chemokines and chemoattractants in vivo on leukocyte dynamics. Immunol. Rev..

[144] de Oliveira S., Rosowski E.E., Huttenlocher A. (2016). Neutrophil migration in infection and wound repair: Going forward in reverse. Nat. Rev. Immunol..

[145] Corasolla Carregari V., Stuani Floriano R., Rodrigues-Simioni L., Winck F.V., Baldasso P.A., Ponce-Soto L.A., Marangoni S. (2013). Biochemical, Pharmacological, and Structural Characterization of New Basic Bbil-TX from *Bothriopsis bilineata* Snake Venom. Biomed Res. Int..

[146] Moura A.A.D., Kayano A.M., Oliveira G.A., Setúbal S.S., Ribeiro J.G., Barros N.B., Nicolete R., Moura L.A., Fuly A.L., Nomizo A. (2014). Purification and Biochemical Characterization of Three Myotoxins from *Bothrops mattogrossensis* Snake Venom with Toxicity against Leishmania and Tumor Cells. Biomed Res. Int..

[147] Boeno C.N., Paloschi M.V., Lopes J.A., Pires W.L., Setúbal S.D.S., Evangelista J.R., Soares A.M., Zuliani J.P. (2019). Inflammasome Activation Induced by a Snake Venom Lys49-Phospholipase A2 Homologue. Toxins.

[148] Ranéia e Silva P.A., de Lima D.S., Mesquita Luiz J.P., Câmara N.O.S., Alves-Filho J.C.F., Pontillo A., Bortoluci K.R., Faquim-Mauro E.L. (2021). Inflammatory effect of Bothropstoxin-I from Bothrops jararacussu venom mediated by NLRP3 inflammasome involves ATP and P2X7 receptor. Clin. Sci..

[149] Setúbal S.S., Pontes A.S., Furtado J.L., Xavier C.V., Silva F.L., Kayano A.M., Izidoro L.F.M., Soares A.M., Calderon L.A., Stábeli R.G. (2013). Action of two phospholipases A2 purified from *Bothrops alternatus* snake venom on macrophages. Biochemistry.

[150] Rueda A.Q., Rodríguez I.G., Arantes E.C., Setúbal S.S., Calderon L.D.A., Zuliani J.P., Stábeli R.G., Soares A.M. (2013). Biochemical Characterization, Action on Macrophages, and Superoxide Anion Production of Four Basic Phospholipases A 2 from Panamanian *Bothrops asper* Snake Venom. Biomed Res. Int..

[151] Zuliani J.P., Gutiérrez J.M., e Silva L.L.C., Sampaio S.C., Lomonte B., de Fátima Pereira Teixeira C. (2005). Activation of cellular functions in macrophages by venom secretory Asp-49 and Lys-49 phospholipases A2. Toxicon.

[152] Furtado J.L., Oliveira G.A., Pontes A.S., Setúbal S.D.S., Xavier C.V., Lacouth-Silva F., Lima B.F., Zaqueo K.D., Kayano A.M., Calderon L.A. (2014). Activation of J77A.1 macrophages by three phospholipases A2 isolated from *Bothrops atrox* snake venom. Biomed Res. Int..

[153] Brinkmann V., Zychlinsky A. (2007). Beneficial suicide: Why neutrophils die to make NETs. Nat. Rev. Microbiol..

[154] Castanheira F.V.S., Kubes P. (2019). Neutrophils and NETs in modulating acute and chronic inflammation. Blood.

[155] Setúbal S.D.S., Pontes A.S., Nery N.M., Rego C.M.A., Santana H.M., de Lima A.M., Boeno C.N., Paloschi M.V., Soares A.M., Zuliani J.P. (2020). Human neutrophils functionality under effect of an Asp49 phospholipase A2 isolated from *Bothrops atrox* venom. Toxicon X.

[156] Bon C., Choumet V., Delot E., Faure G., Robbe-Vincent A., Saliou B. (1994). Different Evolution of Phospholipase A 2 Neurotoxins (Beta-Neurotoxins) from Elapidae and Viperidae Snakes. Ann. N. Y. Acad. Sci..

[157] Rossetto O., Morbiato L., Caccin P., Rigoni M., Montecucco C. (2006). Presynaptic enzymatic neurotoxins. J. Neurochem..

[158] Sampaio S.C., Brigatte P., Sousa-E-Silva M.C.C., Dos-Santos E.C., Rangel-Santos A.C., Curi R., Cury Y. (2003). Contribution of crotoxin for the inhibitory effect of Crotalus durissus terrificus snake venom on macrophage function. Toxicon.

[159] Sampaio S.C., Rangel-Santos A.C., Peres C.M., Curi R., Cury Y. (2005). Inhibitory effect of phospholipase A2 isolated from Crotalus durissus terrificus venom on macrophage function. Toxicon.

[160] Freitas A.P., Favoretto B.C., Clissa P.B., Sampaio S.C., Faquim-Mauro E.L. (2018). Crotoxin Isolated from Crotalus durissus terrificus Venom Modulates the Functional Activity of Dendritic Cells via Formyl Peptide Receptors. J. Immunol. Res..

[161] Giannotti K.C., Leiguez E., De Carvalho A.E.Z., Nascimento N.G., Matsubara M.H., Fortes-Dias C.L., Moreira V., Teixeira C. (2017). A snake venom group IIA PLA2 with immunomodulatory activity induces formation of lipid droplets containing 15-d-PGJ2 in macrophages. Sci. Rep..

[162] Moreira V., De Castro Souto P.C.M., Ramirez Vinolo M.A., Lomonte B., María Gutiérrez J., Curi R., Teixeira C. (2013). A catalytically-inactive snake venom Lys49 phospholipase A2 homolog induces expression of cyclooxygenase-2 and production of prostaglandins through selected signaling pathways in macrophages. Eur. J. Pharmacol..

[163] Moreira V., Gutiérrez J.M., Amaral R.B., Zamunér S.R., de Fátima Pereira Teixeira C. (2009). Effects of *Bothrops asper* snake venom on the expression of cyclooxygenases and production of prostaglandins by peritoneal leukocytes in vivo, and by isolated neutrophils and macrophages in vitro. Prostaglandins Leukot. Essent. Fat. Acids.

[164] Moreira V., Lomonte B., Vinolo M.A.R., Curi R., Gutiérrez J.M., Teixeira C. (2014). An asp49 phospholipase A2 from snake venom induces cyclooxygenase-2 expression and prostaglandin E2 production via activation of NF- κ B, p38MAPK, and PKC in macrophages. Mediat. Inflamm..

[165] Moreira V., Gutiérrez J.M., Amaral R.B., Lomonte B., Purgatto E., Teixeira C. (2011). A phospholipase A2 from *Bothrops asper* snake venom activates neutrophils in culture: Expression of cyclooxygenase-2 and PGE2 biosynthesis. Toxicon.

[166] Moreira V., Gutiérrez J.M., Lomonte B., Vinolo M.A.R., Curi R., Lambeau G., Teixeira C. (2020). 12-HETE is a regulator of PGE2 production via COX-2 expression induced by a snake venom group IIA phospholipase A2 in isolated peritoneal macrophages. Chem. Biol. Interact..

[167] Cristina Giannotti K., Leiguez E., Moreira V., Nascimento N.G., Lomonte B., Gutiérrez J.M., Lopes De Melo R., Teixeira C. (2013). A Lys49 phospholipase A2, isolated from *Bothrops asper* snake venom, induces lipid droplet formation in macrophages which depends on distinct signaling pathways and the C-terminal region. Biomed Res. Int..

[168] Giannotti K.C., Weinert S., Viana M.N., Leiguez E., Araujo T.L.S., Laurindo F.R.M., Lomonte B., Braun-Dullaeus R., Teixeira C. (2019). A secreted phospholipase A2 induces formation of smooth muscle foam cells which transdifferentiate to macrophage-like state. Molecules.

[169] Leiguez E., Motta P., Maia Marques R., Lomonte B., Sampaio S.V., Teixeira C. (2020). A Representative GIIA Phospholipase A2 Activates Preadipocytes to Produce Inflammatory Mediators Implicated in Obesity Development. Biomolecules.

[170] Leiguez E., Giannotti K.C., Do Nascimento Viana M., Matsubara M.H., Fernandes C.M., Gutiérrez J.M., Lomonte B., Teixeira C. (2018). A snake venom-secreted phospholipase A2 induces foam cell formation depending on the activation of factors involved in lipid homeostasis. Mediat. Inflamm..

[171] Murakami M., Nakatani Y., Atsumi G.I., Inoue K., Kudo I. (2017). Regulatory functions of phospholipase A2. Crit. Rev. Immunol..

[172] Yokomizo T., Ogawa Y., Uozumi N., Kume K., Izumi T., Shimizu T. (1997). cDNA Cloning and Mutagenesis Study of Leukotriene B4 12-Hydroxydehydrogenase. Adv. Exp. Med. Biol..

[173] Sarau H.M., Foley J.J., Schmidt D.B., Martin L.D., Webb E.F., Tzimas M.N., Breton J.J., Chabot-Fletcher M., Underwood D.C., Hay D.W.P. (1999). In vitro and in vivo pharmacological characterization of SB 201993, an eicosanoid-like LTB4receptor antagonist with anti-inflammatory activity. Prostaglandins Leukot. Essent. Fat. Acids.

[174] Serhan C.N., Takano T., Maddox J.F. (1999). Aspirin-Triggered 15-Epi-Lipoxin A4 and Stable Analogs of Lipoxin A4 are Potent Inhibitors of Acute Inflammation. Adv. Exp. Med. Biol..

[175] Chen J.-K., Wang D.-W., Falck J.R., Capdevila J., Harris R.C. (1999). Transfection of an Active Cytochrome P450 Arachidonic Acid Epoxygenase Indicates That 14,15-Epoxyeicosatrienoic Acid Functions as an Intracellular Second Messenger in Response to Epidermal Growth Factor. J. Biol. Chem..

[176] Calder P.C. (2020). Eicosanoids. Essays Biochem..

[177] Samuelsson B., Dahlén S.-E., Lindgren J.Å., Rouzer C.A., Serhan C.N. (1987). Leukotrienes and Lipoxins: Structures, Biosynthesis, and Biological Effects. Science.

[178] Rocca B., FitzGerald G.A. (2002). Cyclooxygenases and prostaglandins: Shaping up the immune response. Int. Immunopharmacol..

[179] O’Neill G.P., Ford-Hutchinson A.W. (1993). Expression of mRNA for cyclooxygenase-1 and cyclooxygenase-2 in human tissues. FEBS Lett..

[180] Merlie J.P., Fagan D., Mudd J., Needleman P. (1988). Isolation and characterization of the complementary DNA for sheep seminal vesicle prostaglandin endoperoxide synthase (cyclooxygenase). J. Biol. Chem..

[181] Funk C.D., Funk L.B., Kennedy M.E., Pong A.S., Fitzgerald G.A. (1991). Human platelet/erythroleukemia cell prostaglandin G/H synthase: cDNA cloning, expression, and gene chromosomal assignment. FASEB J..

[182] Pannunzio A., Coluccia M. (2018). Cyclooxygenase-1 (COX-1) and COX-1 inhibitors in cancer: A review of oncology and medicinal chemistry literature. Pharmaceuticals.

[183] Pruzanski W., Stefanski E., Vadas P., Kennedy B.P., van den Bosch H. (1998). Regulation of the cellular expression of secretory and cytosolic phospholipases A2, and cyclooxygenase-2 by peptide growth factors. Biochim. Biophys. Acta-Mol. Cell Res..

[184] Martínez-Colón G.J., Moore B.B. (2018). Prostaglandin E2 as a Regulator of Immunity to Pathogens. Pharmacol. Ther..

[185] Minghetti L. (2004). Cyclooxygenase-2 (COX-2) in Inflammatory and Degenerative Brain Diseases. J. Neuropathol. Exp. Neurol..

[186] Zidar N., Odar K., Glavac D., Jerse M., Zupanc T., Stajer D. (2009). Cyclooxygenase in normal human tissues—Is COX-1 really a constitutive isoform, and COX-2 an inducible isoform?. J. Cell. Mol. Med..

[187] Moreira V., Gutiérrez J.M., Soares A.M., Zamunér S.R., Purgatto E., de Fátima Pereira Teixeira C. (2008). Secretory phospholipases A2 isolated from *Bothrops asper* and from Crotalus durissus terrificus snake venoms induce distinct mechanisms for biosynthesis of prostaglandins E2 and D2 and expression of cyclooxygenases. Toxicon.

[188] Gerritsen M.E. (1996). Physiological and pathophysiological roles of eicosanoids in the microcirculation. Cardiovasc. Res..

[189] Kida T., Sawada K., Kobayashi K., Hori M., Ozaki H., Murata T. (2014). Diverse effects of prostaglandin e2on vascular contractility. Heart Vessel.

[190] Gutiérrez J., Lomonte B., Chaves F., Moreno E., Cerdas L. (1986). Pharmacological activities of a toxic phospholipase a isolated from the venom of the snake *Bothrops asper*. Comp. Biochem. Physiol. Part C Comp. Pharmacol..

[191] Moreira V., Zamuner S.R., Wallace J.L., de Fátima PereiraTeixeira C. (2007). Bothrops jararaca and Crotalus durissus terrificus venoms elicit distinct responses regarding to production of prostaglandins E2 and D2, and expression of cyclooxygenases. Toxicon.

[192] Kini R.M., Evans H.J. (1995). The role of enzymatic activity in inhibition of the extrinsic tenase complex by phospholipase A2 isoenzymes from Naja nigricollis venom. Toxicon.

[193] Thommesen L., Sjursen W., Gåsvik K., Hanssen W., Brekke O.L., Skattebøl L., Holmeide A.K., Espevik T., Johansen B., Laegreid A. (1998). Selective inhibitors of cytosolic or secretory phospholipase A2 block TNF-induced activation of transcription factor nuclear factor-kappa B and expression of ICAM-1. J. Immunol..

[194] Anthonsen M.W., Solhaug A., Johansen B. (2001). Functional Coupling between Secretory and Cytosolic Phospholipase A2 Modulates Tumor Necrosis Factor-α- and Interleukin-1β-induced NF-κB Activation. J. Biol. Chem..

[195] Balsinde J., Balboa M.A., Dennis E.A. (1998). Functional coupling between secretory phospholipase A2 and cyclooxygenase-2 and its regulation by cytosolic group IV phospholipase A2. Proc. Natl. Acad. Sci. USA.

[196] Peng Z., Chang Y., Fan J., Ji W., Su C. (2021). Phospholipase A2 superfamily in cancer. Cancer Lett..

[197] Naraba H., Murakami M., Matsumoto H., Shimbara S., Ueno A., Kudo I., Oh-ishi S. (1998). Segregated coupling of phospholipases A2, cyclooxygenases, and terminal prostanoid synthases in different phases of prostanoid biosynthesis in rat peritoneal macrophages. J. Immunol..

[198] Kuwata H., Nakatani Y., Murakami M., Kudo I. (1998). Cytosolic Phospholipase A2 Is Required for Cytokine-induced Expression of Type IIA Secretory Phospholipase A2 That Mediates Optimal Cyclooxygenase-2-dependent Delayed Prostaglandin E2 Generation in Rat 3Y1 Fibroblasts. J. Biol. Chem..

[199] Murakami M., Shimbara S., Kambe T., Kuwata H., Winstead M.V., Tischfield J.A., Kudo I. (1998). The Functions of Five Distinct Mammalian Phospholipase A2s in Regulating Arachidonic Acid Release. J. Biol. Chem..

[200] Murakami M., Das S., Kim Y.-J., Cho W., Kudo I. (2003). Perinuclear localization of cytosolic phospholipase A 2 α is important but not obligatory for coupling with cyclooxygenases. FEBS Lett..

[201] Ghosh M., Stewart A., Tucker D.E., Bonventre J.V., Murphy R.C., Leslie C.C. (2004). Role of Cytosolic Phospholipase A 2 in Prostaglandin E 2 Production by Lung Fibroblasts. Am. J. Respir. Cell Mol. Biol..

[202] Belich M.P., Salmerón A., Johnston L.H., Ley S.C. (1999). TPL-2 kinase regulates the proteolysis of the NF-κB-inhibitory protein NF-κB1 p105. Nature.

[203] Kifor O., MacLeod R.J., Diaz R., Bai M., Yamaguchi T., Yao T., Kifor I., Brown E.M. (2001). Regulation of MAP kinase by calcium-sensing receptor in bovine parathyroid and CaR-transfected HEK293 cells. Am. J. Physiol. Physiol..

[204] Aoki K., Zubkov A.Y., Parent A.D., Zhang J.H. (2000). Mechanism of ATP-Induced [Ca^2+^] i Mobilization in Rat Basilar Smooth Muscle Cells. Stroke.

[205] Leiguez E., Giannotti K.C., Moreira V., Matsubara M.H., Gutíerrez J.M., Lomonte B., Rodriǵuez J.P., Balsinde J., Teixeira C. (2014). Critical role of TLR2 and MyD88 for functional response of macrophages to a group IIA-secreted phospholipase A2from snake venom. PLoS ONE.

[206] Soto M.E., Guarner-Lans V., Herrera-Morales K.Y., Pérez-Torres I. (2018). Participation of arachidonic acid metabolism in the aortic aneurysm formation in patients with Marfan syndrome. Front. Physiol..

[207] Rikitake Y., Hirata K., Kawashima S., Takeuchi S., Shimokawa Y., Kojima Y., Inoue N., Yokoyama M. (2001). Signaling Mechanism Underlying COX-2 Induction by Lysophosphatidylcholine. Biochem. Biophys. Res. Commun..

[208] Hughes-Fulford M., Tjandrawinata R.R., Li C.-F., Sayyah S. (2005). Arachidonic acid, an omega-6 fatty acid, induces cytoplasmic phospholipase A 2 in prostate carcinoma cells. Carcinogenesis.

[209] Ruipérez V., Casas J., Balboa M.A., Balsinde J. (2007). Group V Phospholipase A 2 -Derived Lysophosphatidylcholine Mediates Cyclooxygenase-2 Induction in Lipopolysaccharide-Stimulated Macrophages. J. Immunol..

[210] Gubern A., Barceló-Torns M., Casas J., Barneda D., Masgrau R., Picatoste F., Balsinde J., Balboa M.A., Claro E. (2009). Lipid droplet biogenesis induced by stress involves triacylglycerol synthesis that depends on Group VIA phospholipase A2. J. Biol. Chem..

[211] Guijas C., Pérez-Chacón G., Astudillo A.M., Rubio J.M., Gil-de-Gómez L., Balboa M.A., Balsinde J. (2012). Simultaneous activation of p38 and JNK by arachidonic acid stimulates the cytosolic phospholipase A2-dependent synthesis of lipid droplets in human monocytes. J. Lipid Res..

[212] Jarc E., Kump A., Malavašič P., Eichmann T.O., Zimmermann R., Petan T. (2018). Lipid droplets induced by secreted phospholipase A2 and unsaturated fatty acids protect breast cancer cells from nutrient and lipotoxic stress. Biochim. Biophys. Acta-Mol. Cell Biol. Lipids.

[213] Arrese E.L., Saudale F.Z., Soulages J.L. (2014). Lipid droplets as signaling platforms linking metabolic and cellular functions. Lipid Insights.

[214] Onal G., Kutlu O., Gozuacik D., Dokmeci Emre S. (2017). Lipid Droplets in Health and Disease. Lipids Health Dis..

[215] Welte M.A. (2015). Expanding roles for lipid droplets. Curr. Biol..

[216] Bosch M., Sánchez-Álvarez M., Fajardo A., Kapetanovic R., Steiner B., Dutra F., Moreira L., López J.A., Campo R., Marí M. (2020). Mammalian lipid droplets are innate immune hubs integrating cell metabolism and host defense. Science.

[217] Karagiannis F., Masouleh S.K., Wunderling K., Surendar J., Schmitt V., Kazakov A., Michla M., Hölzel M., Thiele C., Wilhelm C. (2020). Lipid-Droplet Formation Drives Pathogenic Group 2 Innate Lymphoid Cells in Airway Inflammation. Immunity.

[218] Marschallinger J., Iram T., Zardeneta M., Lee S.E., Lehallier B., Haney M.S., Pluvinage J.V., Mathur V., Hahn O., Morgens D.W. (2020). Lipid-droplet-accumulating microglia represent a dysfunctional and proinflammatory state in the aging brain. Nat. Neurosci..

[219] Garces F., López F., Nĩo C., Fernandez A., Chacin L., Hurt-Camejo E., Camejo G., Apitz-Castro R. (2010). High plasma phospholipase A 2 activity, inflammation markers, and LDL alterations in obesity with or without type 2 diabetes. Obesity.

[220] Tall A.R., Yvan-Charvet L. (2015). Cholesterol, inflammation and innate immunity. Nat. Rev. Immunol..

[221] Schaftenaar F., Frodermann V., Kuiper J., Lutgens E. (2016). Atherosclerosis: The interplay between lipids and immune cells. Curr. Opin. Lipidol..

[222] Rodríguez J.P., Leiguez E., Guijas C., Lomonte B., Gutiérrez J.M., Teixeira C., Balboa M.A., Balsinde J. (2020). A lipidomic perspective of the action of group iia secreted phospholipase a2 on human monocytes: Lipid droplet biogenesis and activation of cytosolic phospholipase a2α. Biomolecules.

[223] Moujaber O., Stochaj U. (2020). The Cytoskeleton as Regulator of Cell Signaling Pathways. Trends Biochem. Sci..

[224] Leiguez E., Zuliani J.P., Cianciarullo A.M., Fernandes C.M., Gutierrez J.M., Teixeira C. (2011). A group IIA-secreted phospholipase A2 from snake venom induces lipid body formation in macrophages: The roles of intracellular phospholipases A2 and distinct signaling pathways. J. Leukoc. Biol..

[225] Yu Y.H., Liao P.R., Guo C.J., Chen C.H., Mochly-Rosen D., Chuang L.M. (2016). PKC-ALDH2 pathway plays a novel role in adipocyte differentiation. PLoS ONE.

[226] Huang S.H., Shen W.J., Yeo H.L., Wang S.M. (2004). Signaling pathway of magnolol-stimulated lipolysis in sterol ester-loaded 3T3-L1 preadipocyes. J. Cell. Biochem..

[227] Le Lay S., Hajduch E., Lindsay M.R., Le Lièpvre X., Thiele C., Ferré P., Parton R.G., Kurzchalia T., Simons K., Dugail I. (2006). Cholesterol-induced caveolin targeting to lipid droplets in adipocytes: A role for caveolar endocytosis. Traffic.

[228] Than N.G., Sumegi B., Bellyei S., Berki T., Szekeres G., Janaky T., Szigeti A., Bohn H., Than G.N. (2003). Lipid droplet and milk lipid globule membrane associated placental protein 17b (PP17b) is involved in apoptotic and differentiation processes of human epithelial cervical carcinoma cells. Eur. J. Biochem..

[229] Zhong W., Fan B., Cong H., Wang T., Gu J. (2019). Oleic acid-induced perilipin 5 expression and lipid droplets formation are regulated by the PI3K/PPARα pathway in HepG2 cells. Appl. Physiol. Nutr. Metab..

[230] Han X., Wang T., Zhang J., Liu X., Li Z., Wang G., Song Q., Pang D., Ouyang H., Tang X. (2015). Apolipoprotein CIII regulates lipoprotein-associated phospholipase A2 expression via the MAPK and NFκB pathways. Biol. Open.

[231] Hu S.-B., Zou Q., Lv X., Zhou R.L., Niu X., Weng C., Chen F., Fan Y.W., Deng Z.Y., Li J. (2020). 9t18:1 and 11t18:1 activate the MAPK pathway to regulate the expression of PLA2 and cause inflammation in HUVECs. Food Funct..

[232] Andersson L., Boström P., Ericson J., Rutberg M., Magnusson B., Marchesan D., Ruiz M., Asp L., Huang P., Frohman M.A. (2006). PLD1 and ERK2 regulate cytosolic lipid droplet formation. J. Cell Sci..

[233] Boström P., Andersson L., Li L., Perkins R., Højlund K., Borén J., Olofsson S.O. (2009). The assembly of lipid droplets and its relation to cellular insulin sensitivity. Biochem. Soc. Trans..

[234] Reustle A., Torzewski M. (2018). Role of p38 MAPK in atherosclerosis and aortic valve sclerosis. Int. J. Mol. Sci..

[235] Kavurma M.M., Rayner K.J., Karunakaran D. (2017). The walking dead: Macrophage inflammation and death in atherosclerosis. Curr. Opin. Lipidol..

[236] Chakraborti S. (2003). Phospholipase A2 isoforms: A perspective. Cell. Signal..

[237] Hooks S.B., Cummings B.S. (2008). Role of Ca2+-independent phospholipase A2 in cell growth and signaling. Biochem. Pharmacol..

[238] Kita Y., Shindou H., Shimizu T. (2019). Cytosolic phospholipase A 2 and lysophospholipid acyltransferases. Biochim. Biophys. Acta-Mol. Cell Biol. Lipids.

[239] Grygiel-Górniak B. (2014). Peroxisome proliferator-activated receptors and their ligands: Nutritional and clinical implications—A review. Nutr. J..

[240] Engin A.B., Engin A. (2017). Obesity and Lipotoxicity.

[241] Mei C.-L., He P., Cheng B., Liu W., Wang Y.-F., Wan J.-J. (2009). Chlamydia pneumoniae induces macrophage-derived foam cell formation via PPAR α and PPAR γ-dependent pathways. Cell Biol. Int..

[242] Chistiakov D.A., Melnichenko A.A., Myasoedova V.A., Grechko A.V., Orekhov A.N. (2017). Mechanisms of foam cell formation in atherosclerosis. J. Mol. Med..

[243] Son S.H., Goo Y.H., Chang B.H., Paul A. (2012). Perilipin 2 (PLIN2)-deficiency does not increase cholesterol-induced toxicity in macrophages. PLoS ONE.

[244] Turkish A., Sturley S.L. (2007). Regulation of Triglyceride Metabolism. I. Eukaryotic neutral lipid synthesis: “Many ways to skin ACAT or a DGAT”. Am. J. Physiol.-Gastrointest. Liver Physiol..

[245] Yu X.H., Fu Y.C., Zhang D.W., Yin K., Tang C.K. (2013). Foam cells in atherosclerosis. Clin. Chim. Acta.

[246] Cruz A.L.S., Barreto E.D.A., Fazolini N.P.B., Viola J.P.B., Bozza P.T. (2020). Lipid droplets: Platforms with multiple functions in cancer hallmarks. Cell Death Dis..

[247] Dias S.S.G., Soares V.C., Ferreira A.C., Sacramento C.Q., Fintelman-Rodrigues N., Temerozo J.R., Teixeira L., Nunes da Silva M.A., Barreto E., Mattos M. (2020). Lipid droplets fuel SARS-CoV-2 replication and production of inflammatory mediators. PLoS Pathog..

[248] Jarc E., Petan T. (2020). A twist of FATe: Lipid droplets and inflammatory lipid mediators. Biochimie.

[249] De Carvalho A.E.Z., Giannotti K., Junior E.L., Matsubara M., Dos Santos M.C., Fortes-Dias C.L., Teixeira C. (2019). Crotalus durissus ruruima Snake Venom and a Phospholipase A2 Isolated from This Venom Elicit Macrophages to Form Lipid Droplets and Synthesize Inflammatory Lipid Mediators. J. Immunol. Res..

[250] Juge-Aubry C.E., Henrichot E., Meier C.A. (2005). Adipose tissue: A regulator of inflammation. Best Pract. Res. Clin. Endocrinol. Metab..

[251] Oikonomou E.K., Antoniades C. (2019). The role of adipose tissue in cardiovascular health and disease. Nat. Rev. Cardiol..

[252] La Cava A., Matarese G. (2004). The weight of leptin in immunity. Nat. Rev. Immunol..

[253] Tsatsanis C., Zacharioudaki V., Androulidaki A., Dermitzaki E., Charalampopoulos I., Minas V., Gravanis A., Margioris A.N. (2005). Adiponectin induces TNF-α and IL-6 in macrophages and promotes tolerance to itself and other pro-inflammatory stimuli. Biochem. Biophys. Res. Commun..

[254] Yamaguchi N., Argueta J.G.M., Masuhiro Y., Kagishita M., Nonaka K., Saito T., Hanazawa S., Yamashita Y. (2005). Adiponectin inhibits Toll-like receptor family-induced signaling. FEBS Lett..

[255] Martínez-Sánchez N. (2020). There and back again: Leptin actions in white adipose tissue. Int. J. Mol. Sci..

